# Setosphlides A–D, New Isocoumarin Derivatives from the Entomogenous Fungus *Setosphaeria rostrate* LGWB-10

**DOI:** 10.1007/s13659-020-00292-8

**Published:** 2021-01-07

**Authors:** Wenbin Gao, Xiaoxia Wang, Fengli Chen, Chunqing Li, Fei Cao, Duqiang Luo

**Affiliations:** College of Life Science, Institute of Life Science and Green Development, Key Laboratory of Medicinal Chemistry and Molecular Diagnosis of Ministry of Education, Hebei University, Baoding, 071002 China

**Keywords:** Entomogenous fungus, *Setosphaeria rostrate*, Isocoumarin, Absolute configuration

## Abstract

**Abstract:**

Investigation of the entomogenous fungus *Setosphaeria rostrate* LGWB-10 from *Harmonia axyridis* led to the isolation of four new isocoumarin derivatives, setosphlides A–D (**1**–**4**), and four known analogues (**5**–**8**). Their planar structures and the relative configurations were elucidated by comprehensive spectroscopic methods. The absolute configurations of isocoumarin nucleus for **1**–**4** were elucidated by their ECD spectra. The C-10 relative configurations for the pair of C-10 epimers (**1** and **2**) were established by comparing the magnitude of the computed ^13^C NMR chemical shifts (Δ*δ*_calcd._) with the experimental ^13^C NMR values (Δ*δ*_exp._) for the epimers. All of the isolated compounds (**1**–**8**) were evaluated for their cytotoxicities against four human tumor cell lines MCF-7, MGC-803, HeLa, and Huh-7.

**Graphic Abstract:**

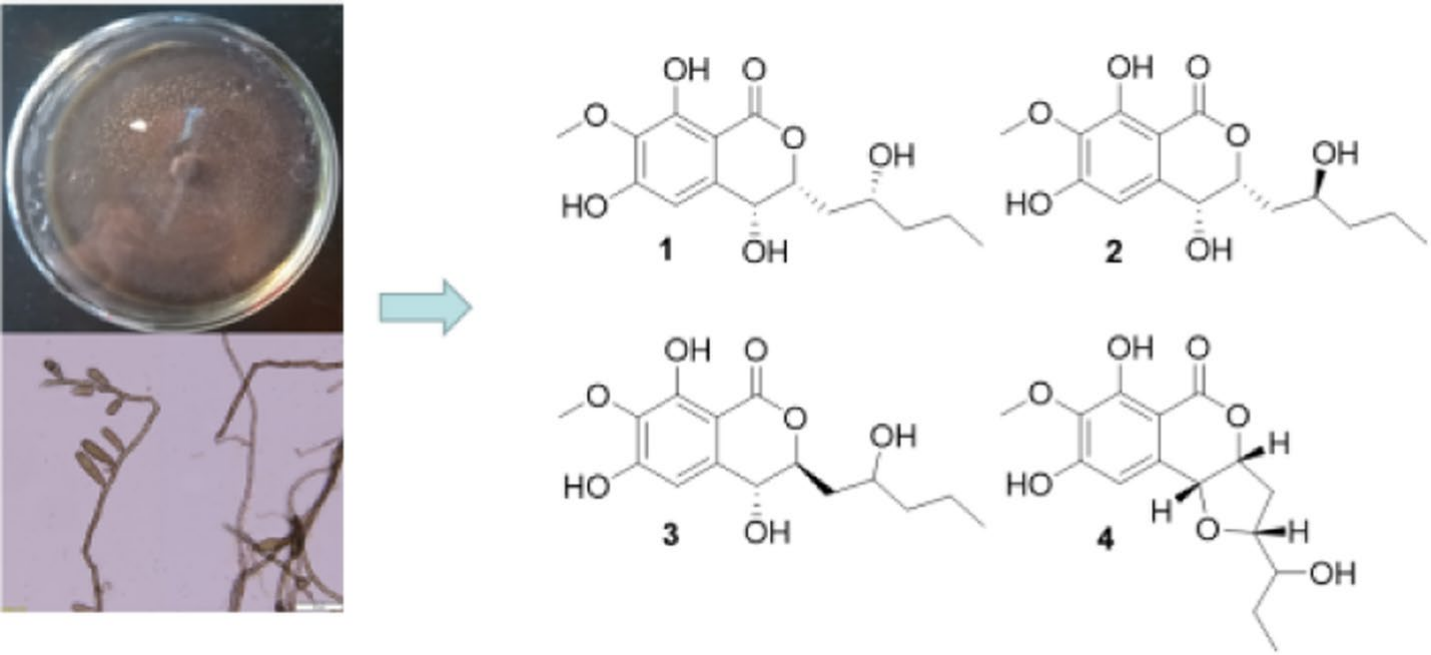

**Supplementary Information:**

The online version of this article (10.1007/s13659-020-00292-8) contains supplementary material, which is available to authorized users.

## Introduction

**Fig. 1:**
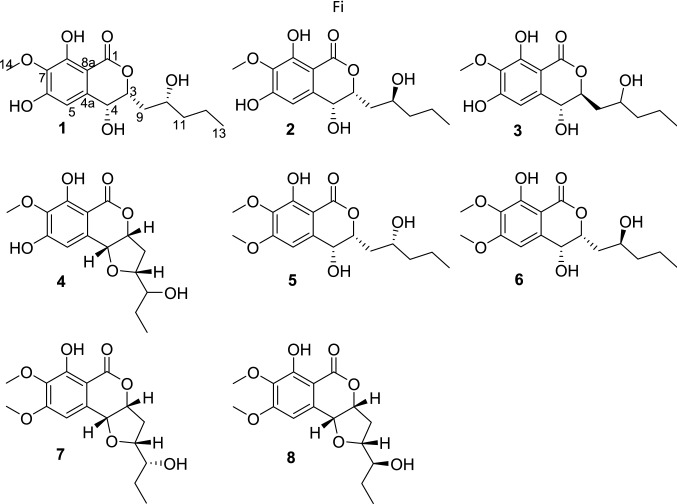
The chemical structures of compounds **1**–**8**

Symbiosic microorganisms from insects, which are well-known as a rich source of bioactive natural products, have attracted widespread attention [1–3]. Especially, due to the special environmental conditions, the bioactive natural products from symbiotic microorganisms as a rich source of various compounds with complex structures and excellent activities, may reshape the experts’ views on the drug ability of natural products [4]. Among them, isocoumarin derivatives have been isolated as antifungal, insecticidal, and phytotoxic secondary metabolites from several fungal sources [5]. However, it made such a task extremely challenging to assign their absolute configurations, when a side chain attached to isocoumarin derivative nuclear, as the high free rotation of the stereogenic centers in chains [6]. During our ongoing search for bioactive compounds from fungi, *Setosphaeria rostrate* LGWB-10 was selected for chemical exploration based on HPLC–DAD and HPLC–MS analyses of its EtOAc extract. Subsequently, eight isocoumarin derivatives, including four new setosphlides A–D (**1**–**4**) and four known analogues, (3*R*,4*R*)-4,8-dihydroxy-3-((*R*)-2-hydroxypentyl)-6,7-dimethoxyisochroman-1-one (**5**) [5], (3*R*,4*R*)-4,8-dihydroxy-3-((*R*)-2-hydroxypentyl)-6,7-dimethoxyisochroman-1-one (**6**) [5], (12*R*)-12-hydroxymonocerin (**7**) [7], and (12*S*)-12-hydroxymonocerin (**8**) [6] (Fig. 1) were isolated from *Setosphaeria rostrate* LGWB-10. Herein, we report the details of the isolation, structure elucidation, absolute configuration determination, and bioactivities of them.

## Results and Discussion

**Fig. 2:**
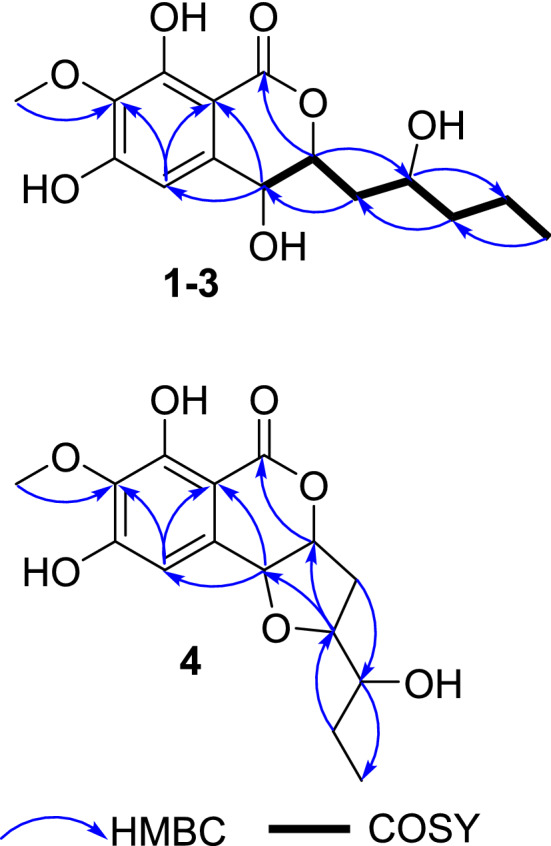
^1^H–^1^H COSY and Key HMBC correlations of **1–4**

Setosphlide A (**1**) was isolated as a colorless oil. The molecular formula of **1** was determined as C_15_H_20_O_7_ by positive HRESIMS data at *m/z* 335.1093 [M + Na]^+^ (calcd. for 335.1101), with six indices of hydrogen deficiency. Analysis of the 1D NMR and HSQC data of **1** (Table 1) revealed the presence of two methyl groups (*δ*_H_ 3.85 and 0.96), three methylene groups (*δ*_H_ 1.50 and 1.42, 2.13 and 1.65, and 1.50), three oxygen-bearing methine groups (*δ*_H_ 4.70, 4.42, and 3.91), and one olefinic proton (*δ*_H_ 6.49). Analysis of ^13^C NMR and HMBC spectra of **1** (Table 2) indicated the presence of 15 carbons, including six quaternary carbons (*δ*_C_ 171.2, 158.5, 157.4, 139.1, 136.3, and 101.5), four methines (*δ*_C_ 108.5, 80.6, 67.8, and 67.7), three methylenes (*δ*_C_ 41.5, 39.5, and 19.9), and two methyls (*δ*_C_ 60.9 and 14.4). Six aromatic carbon signals in the region of *δ*_C_ 101.5–158.5 indicated the existence of a polysubstituted phenyl moiety. All of the proton resonances were assigned to the relevant carbon atoms by the HSQC spectrum. Careful comparison of the ^1^H and ^13^C NMR spectra as well as the MS data of **1** with those of (3*R*,4*R*)-4,8-dihydroxy-3-((*R*)-2-hydroxypentyl)-6,7-dimethoxyisochroman-1-one (**5**) [5], revealed that **1** shared the same scaffold as **5**. The detailed comparison of 1D NMR data between **1** and **5** suggested the 6-OCH_3_ in **5** was absent in **1**, which was confirmed by the key HMBC correlations from H-5 and 7-OCH_3_ to C-4, C-7 and C-15 (Fig. 2). Thus, the planar structure of **1** was assigned.

**Table 1:** ^1^H NMR Data (*δ*) of **1**–**4** (600 MHz, CD_3_OD, J in Hz)

No.	**1**	**2**	**3**	**4**
3	4.70, td (10.2, 1.8)	4.66, td (6.6, 1.8)	4.53, m	5.09, dd (6.0, 3.0)
4	4.42, d (1.8)	4.52, d (1.8)	4.56, m (overlap)	4.59, d (3.0)
5	6.49, s	6.50, s	6.58, s	6.55, s
9	2.13, ddd (14.4, 8.4, 1.8)	2.10, ddd (14.4, 8.4, 4.2)	1.91, ddd (15.0, 10.8, 5.4)	2.55, m
	1.65, ddd (14.4, 10.2, 2.4)	1.97, ddd (14.4, 9.0, 6.6)	1.86, ddd (15.0, 13.2, 6.0)	2.18, dd (14.4, 6.0)
10	3.91, m	3.84, m	3.81, m	4.00, dt (9.0, 6.0)
11	1.50, m	1.52, m	1.48, m	3.43, m
12	1.50, m	1.52, m	1.48, m	1.53, m
	1.42, m	1.47, m	1.36, m	1.39, m
13	0.96, t (7.2)	0.96, t (7.2)	0.96, t (7.2)	0.98, t (7.2)
14	3.85, s	3.85, s	3.81, s	3.83, s

**Table 2:** ^13^C NMR Data (*δ*) of **1**–**4** (150 MHz, CD_3_OD)

No.	**1**	**2**	**3**	**4**
1	171.2, C	171.3, C	170.3, C	169.8, C
3	80.6, CH	81.3, CH	82.9, CH	82.7, CH
4	67.8, CH	66.1, CH	68.2, CH	75.7, C
4a	139.1, C	139.0, C	139.7, C	132.9, C
5	108.5, CH	108.7, CH	107.2, CH	110.5, CH
6	158.5, C	158.5, C	158.8, C	158.4, C
7	136.3, C	136.4, C	135.9, C	136.9, C
8	157.4, C	157.4, C	157.4, C	157.6, C
8a	101.5, C	101.6, C	101.0, C	101.6, C
9	39.5, CH_2_	38.7, CH_2_	40.5, CH_2_	36.8, CH_2_
10	67.7, CH	68.5, CH	69.0, CH	82.8, CH
11	41.5, CH_2_	40.9, CH_2_	40.2, CH_2_	75.6, CH
12	19.9, CH_2_	19.9, CH_2_	19.7, CH_2_	26.9, CH_2_
13	14.4, CH_3_	14.4, CH_3_	14.3, CH_3_	10.5, CH_3_
14	60.9, CH_3_	60.9, CH_3_	60.9, CH_3_	60.9, CH_3_

Setosphlides B and C (**2** and **3**) were obtained with the same molecular formula of **1**. Furthermore, the ^1^H and ^13^C NMR data of **1–3** (Tables 1 and 2) showed striking similarity, suggesting the same structural nucleus of them. In fact, only differences of the NMR signals for CH-3, CH-4, and CH-10 were observed [*δ*_H_ 4.70 (1H, td, *J* = 10.2, 1.8 Hz, H-3), 4.42 (1H, d, *J* = 1.8 Hz, H-4), 3.91 (1H, m, H-10), *δ*_C_ 80.6 (C-3), 67.8 (C-4), and 67.7 (C-10) in **1** vs.* δ*_H_ 4.66 (1H, dt, *J* = 6.6, 1.8 Hz, H-3), 4.52 (1H, d, *J* = 1.8 Hz, H-4), 3.84 (1H, m, H-10), *δ*_C_ 81.3 (C-3), 66.1 (C-4), and 68.5 (C-10) in **2** vs.* δ*_H_ 4.53 (1H, m), 4.56 (1H, m), 3.81 (1H, m, H-10), *δ*_C_ 82.9 (C-3), 68.2 (C-4), and 69.0 (C-10) in **3**], indicating that **1–3** were epimeric isocoumarin derivatives with structural difference at C-3, C-4, and C-10. The above deduction was confirmed by a detailed analysis of the HSQC, ^1^H-^1^H COSY, and HMBC spectra (Fig. 2).

**Fig. 3:**
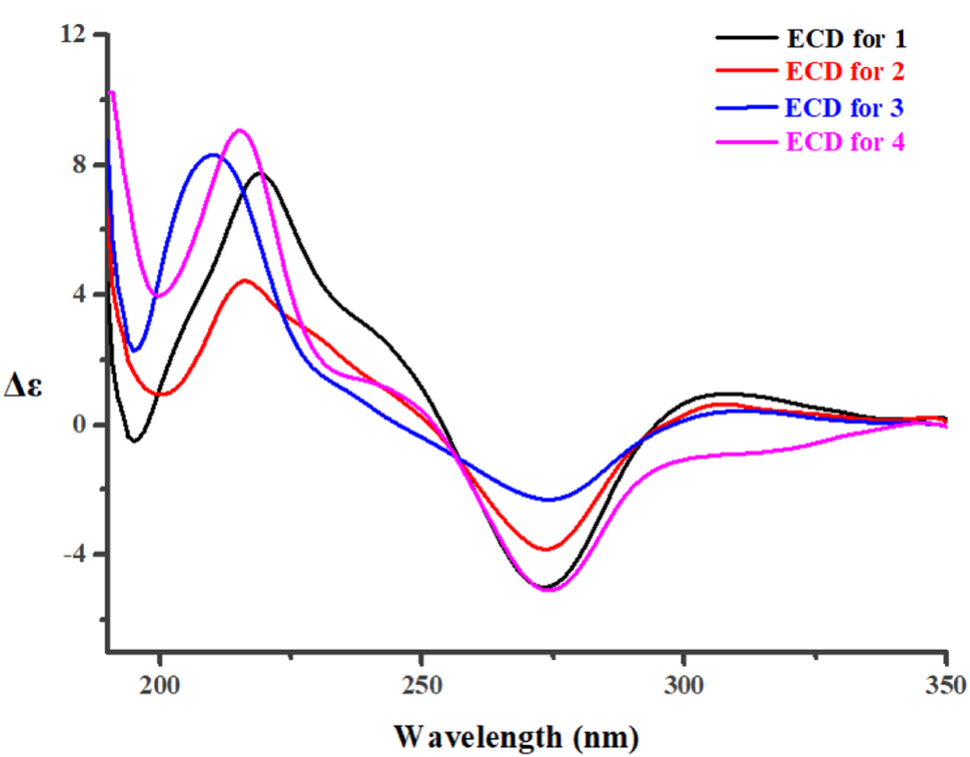
Experimental CD spectra of **1–4**

The relative configurations of C-3 and C-4 in **1**–**3** were determined by their NOESY correlations. For compounds **1** and **2**, NOE correlations from H-3 to H-4 were observed. While, the NOESY correlations between H-4 and H_2_-9 were present for **3**. In order to assign the absolute configurations of C-3 and C-4 in **1**–**3**, electronic circular dichroism (ECD) was carried out for them. The absolute configurations of the C-3 methine carbon in **1**–**3** were deduced by the application of the circular dichroism (CD) exciton chirality method. Further more, According to the earlier references [7, 8], the negative ECD Cotton effect for **1**–**3** around 275 nm (Fig. 3) indicated the 3*R*,4*R*, 3*R*,4*R*, and 3*S*,4*R* configurations for **1**, **2**, and **3**, respecitively.

However, it was difficult to determine the absolute configuration of C-10 in **1**–**3** due to the high conformational flexibility of the chains in them. Especially, the experimental ECD spectra of **1**–**3** were almost identical, indicating that the ECD method had limitations in the assignment of the C-10 absolute configurations for them. Recently, computational methods for atomic chemical shift calculations have been developed and used for the relative configuration identifications of complex natural compounds [9–11]. Compounds **1** and **2** are a pair of epimers with more than one stereogenic carbon. The carbons near C-10 in **1** should have different chemical shifts from those of the corresponding carbons in **2**. Thus, the configurations at C-10 of **1** and **2** could be established by comparing the magnitude of the computed chemical shifts (Δ*δ*_calcd._) for two epimers of (10*R*)-epimer and (10*S*)-epimer [12]. The relative errors (Δ*δ*_calcd._) between the computed ^13^C chemical shifts of (10*R*)-epimer and (10*S*)-epimer, and the relative errors (Δ*δ*_exp._) between experimental ^13^C NMR data of **1** and **2** were summarized in Table 3. Based on the relative error magnitudes (Δ*δ*_calcd._ and Δ*δ*_exp._), the configurations of C-10 for **1** and **2** were suggested to be *R* and *S*, respectively. However, the absolute configuration at C-10 in **4** was undetermined since only one of its C-10 epimer was not obtained.

**Table 3:** Chemical shift differences of selected carbons in 1 and 2

No.	Experimental Δ*δ*_exp_	Calculated Δ*δ*_calcd_
	**1**—**2**	(10*R*)-epimer—(10*S*)-epimer
C-3	–0.7	–0.3
C-4	1.7	2.1
C-10	0.8	0.6
C-11	–0.8	–1.4
C-12	0.6	2.3

Setosphlide D (**4**) was also isolated as a colourless oil with the molecular formula C_15_H_18_O_7_ determined by HRESIMS. The NMR spectra (Tables 1 and 2) of **4** showed a high similarity to those of **1**–**3**. The most significant difference in the ^1^H NMR spectra was the presence of an additional methine signal at *δ*_H_ (3.43, m) in **4**. Furthermore, the key HMBC from H-9 to C-4 indicated that C-4 and C-9 were connected via an oxygen bridge, forming the third ring, a furan C-ring. The relative configuration of C-3, C-4, and C-10 of **4** was determined by NOESY experiment, which showed NOE correlations from H-10 to H-3 and H-4. ECD spectrum suggested the 3*R*,4*R*,10*R* configuration of **4** (Fig. 3).

All of the isolated compounds (**1**–**4**) were evaluated for their cytotoxicities against four human tumor cell lines MCF-7, MGC-803, HeLa, and Huh-7. However, all of the compounds hardly displayed obvious activity (IC_50_ > 200 μM).

## Experimental

### General Experimental Procedures

OR and UV data were acquired on Perkin-Elmer 341 and 241 spectrophotometers, respectively. ECD spectra were measured using a JASCO J-715 spectrometer. 1D and 2D NMR data were recorded on a Bruker AM-600 spectrometer. HRESIMS spectra were recorded on a Bruker apex-ultra 7.0T spectrometer. HPLC was carried out on a Waters 600–2489 with a YMC column (YMC-Pack ODS-A, 250 × 10 mm). Column chromatography (CC) were conducted over silica gel (200–300 mesh) and Sephadex LH-20 gel (25–100 μm). TLC were conducted with silica gel GF_254_ plates.

### Isolation of the Fungal Material

The fungal strain *Setosphaeria rostrate* LGWB-10 was isolated from the *Harmonia axyridis* collected in Baoding, Hebei Province, China. The voucher specimen of the fungus was deposited at College of Life Science of Hebei University with Genbank MN 378541. *Setosphaeria rostrate* was cultured on PDA plate at 28 °C for 7 days, and then inoculated into a 500 mL Erlenmeyer flask containing 200 mL of PDB medium. Flask cultures were incubated at 28 °C on a rotary shaker at 120 rpm/min for 4 days. Solid fermentation was carried out in 100 Erlenmeyer flasks (500 mL), each containing 100 g rice, 80 mL distilled H_2_O, and 5 mL of culture liquid as seed, and incubated at 28 °C for 40 days. The fermented material was extracted three times with methanol (20 L for each time), and the methanol extract was concentrated in vacuo to yield a yellow oily residue (132.6 g). This residue was subjected to silica gel column and eluted with a gradient elution of petroleum ether (PE)/EtOAc (100:0, 90:10, 80:20, 60:40, 50:50, 40:60, 20:80, 10:90, 0:100 (v/v)) to obtain nine fractions Frs.1–9. Among these fractions, Fr.3, eluted with 60% EtOAc–PE (3:2, v:v), was applied to a Sephadex LH-20 CC (CH_2_Cl_2_/MeOH (1:1, v:v)) to remove the pigment to give Fr.3–1 and Fr.3–2. Then, Fr.3–1 was purified by semipreparative HPLC (70% MeOH/H_2_O, 2.0 mL/min) to give **4** (4.2 mg), **7** (6.5 mg), and **8** (6.2 mg). Fr.4 was repeatedly purified by Sephadex LH-20, silica gel CC and semipreparative HPLC to afford compounds **1** (4.1 mg) and **2** (4.3 mg), **3** (3.5 mg), **5** (3.2 mg), and **6** (3.0 mg).

**Setosphlide A (1):** Colorless oil; [*α*]_D_^20^ + 10.2 (*c* 1.00, MeOH); UV (MeOH) *λ*_max_ (log *ε*) 231 (4.67), 274 (0.48), 306 (0.45) nm; ECD (0.50 mM, MeOH) λmax (Δε) 219 (+ 7.76), 273 (− 5.01), 308 (+ 0.95) nm; HRESIMS *m/z* 335.1093 [M + Na]^+^ (calcd for C_15_H_20_O_7_Na, 335.1101). ^1^H and ^13^C NMR data, see Tables 1 and 2.

**Setosphlide B (2):** Colorless oil; [*α*]_D_^20^ + 5.7 (*c* 1.00, MeOH); UV (MeOH) *λ*_max_ (log *ε*) 231 (4.65), 274 (0.43), 307 (0.42) nm; ECD (0.50 mM, MeOH) λmax (Δε) 216 (+ 4.44), 274 (− 3.83), 308 (+ 0.64) nm; HRESIMS *m/z* 311.1090 [M + Na]^+^ (calcd for C_15_H_20_O_7_Na, 335.1101). ^1^H and ^13^C NMR data, see Tables 1 and 2.

**Setosphlide C (3):** Colorless oil; [*α*]_D_^20^ + 20.6 (*c* 1.00, MeOH); UV (MeOH) *λ*_max_ (log *ε*) 232 (4.54), 274 (0.43), 307 (0.44) nm; ECD (0.50 mM, MeOH) λmax (Δε) 210 (+ 24.96), 274 (− 6.90), 311 (+ 1.26) nm; HRESIMS *m/z* 311.1094 [M + Na]^+^ (calcd for C_15_H_20_O_7_Na, 335.1101). ^1^H and ^13^C NMR data, see Tables 1 and 2.

**Setosphlide D (4):** Colorless oil; [*α*]_D_^20^ + 15.3 (*c* 1.00, MeOH); UV (MeOH) *λ*_max_ (log *ε*) 233 (4.54), 275 (2.61), 307 (2.84) nm; ECD (0.50 mM, MeOH) λmax (Δε) 215 (+ 9.07), 277 (− 5.10), 341 (− 0.01) nm; HRESIMS *m/z* 311.1125 [M + H]^+^ (calcd for C_15_H_19_O_7_, 311.1120). ^1^H and ^13^C NMR data, see Tables 1 and 2.

### Computational Section

The molecules of (10*R*)-epimer (**1**) and (10*S*)-epimer (**2**) was constructed and used for conformational searches using the MMFF94S force field by using BARISTA software. A total of 45 stable conformers for **1** and 48 stable conformers for **2** with relative energy within a 10.0 kcal/mol energy window were obtained and optimized at the gas-phase B3LYP/6-311 + G(d) level using the Gaussian 09 package. MPW1PW91 theory at the basis set of B3LYP/6-311 + G(d,p) in the gas phase was applied for ^13^C NMR calculation for (10*R*)-epimer (**1**) and (10*S*)-epimer (**2**).

### Cytotoxity Assay

The cytotoxicities against human breast cancer (MCF-7), human gastric cancer (MGC-803), cervical cancer (HeLa), and human hepatoma (Huh-7) cell lines were evaluated using the MTT method [13]. Cisplatin was used as a positive control.

## Supplementary material

Below is the link to the electronic supplementary material.

Supplementary material 1 (DOCX 2963 KB)
