# Supplementary material for: Setosphlides A–D, New Isocoumarin Derivatives from the Entomogenous Fungus Setosphaeria rostrate LGWB-10
Source: Nat Prod Bioprospect. 2021 Jan 7;11(1):137–42. doi: 10.1007/s13659-020-00292-8 (PMC7933304; doi:10.1007/s13659-020-00292-8)
Supplement: Supplementary file 1 — Supplementary material 1 (DOCX 2963 KB) [file 13659_2020_292_MOESM1_ESM.docx]

**List of Supporting Information**

**Figure S1.** ^1^H NMR (600 MHz, CD_3_OD) spectrum of compound **1**

**Figure S2.** ^13^C NMR (150 MHz, CD_3_OD) spectrum of compound **1**

**Figure S3.** HSQC (CD_3_OD) spectrum of compound **1**

**Figure S4.** COSY (CD_3_OD) spectrum of compound **1**

**Figure S5.** HMBC (CD_3_OD) spectrum of compound **1**

**Figure S6.** NOESY (CD_3_OD) spectrum of compound **1**

**Figure S7.** HRESIMS spectrum of compound **1**

**Figure S8.** ^1^H NMR (600 MHz, CD_3_OD) spectrum of compound **2**

**Figure S9.** ^13^C NMR (150 MHz, CD_3_OD) spectrum of compound **2**

**Figure S10.** HSQC (CD_3_OD) spectrum of compound **2**

**Figure S11.** COSY (CD_3_OD) spectrum of compound **2**

**Figure S12.** HMBC (CD_3_OD) spectrum of compound **2**

**Figure S13.** NOESY (CD_3_OD) spectrum of compound **2**

**Figure S14.** HRESIMS spectrum of compound **2**

**Figure S15.** ^1^H NMR (600 MHz, CD_3_OD) spectrum of compound **3**

**Figure S16.** ^13^C NMR (150 MHz, CD_3_OD) spectrum of compound **3**

**Figure S17.** HSQC (CD_3_OD) spectrum of compound **3**

**Figure S18.** COSY (CD_3_OD) spectrum of compound **3**

**Figure S19.** HMBC (CD_3_OD) spectrum of compound **3**

**Figure S20.** NOESY (CD_3_OD) spectrum of compound **3**

**Figure S21.** HRESIMS spectrum of compound **3**

**Figure S22.** ^1^H NMR (600 MHz, CD_3_OD) spectrum of compound **4**

**Figure S23.** ^13^C NMR (150 MHz, CD_3_OD) spectrum of compound **4**

**Figure S24.** HSQC (CD_3_OD) spectrum of compound **4**

**Figure S25.** COSY (CD_3_OD) spectrum of compound **4**

**Figure S26.** HMBC (CD_3_OD) spectrum of compound **4**

**Figure S27.** NOESY (CD_3_OD) spectrum of compound **4**

**Figure S28.** HRESIMS spectrum of compound **4**

**Table S1.** The coordinate for the lowest-energy conformer (**1**) in ^13^C NMR calculations

**Table S2.** The coordinate for the lowest-energy conformer (**2**) in ^13^C NMR calculations


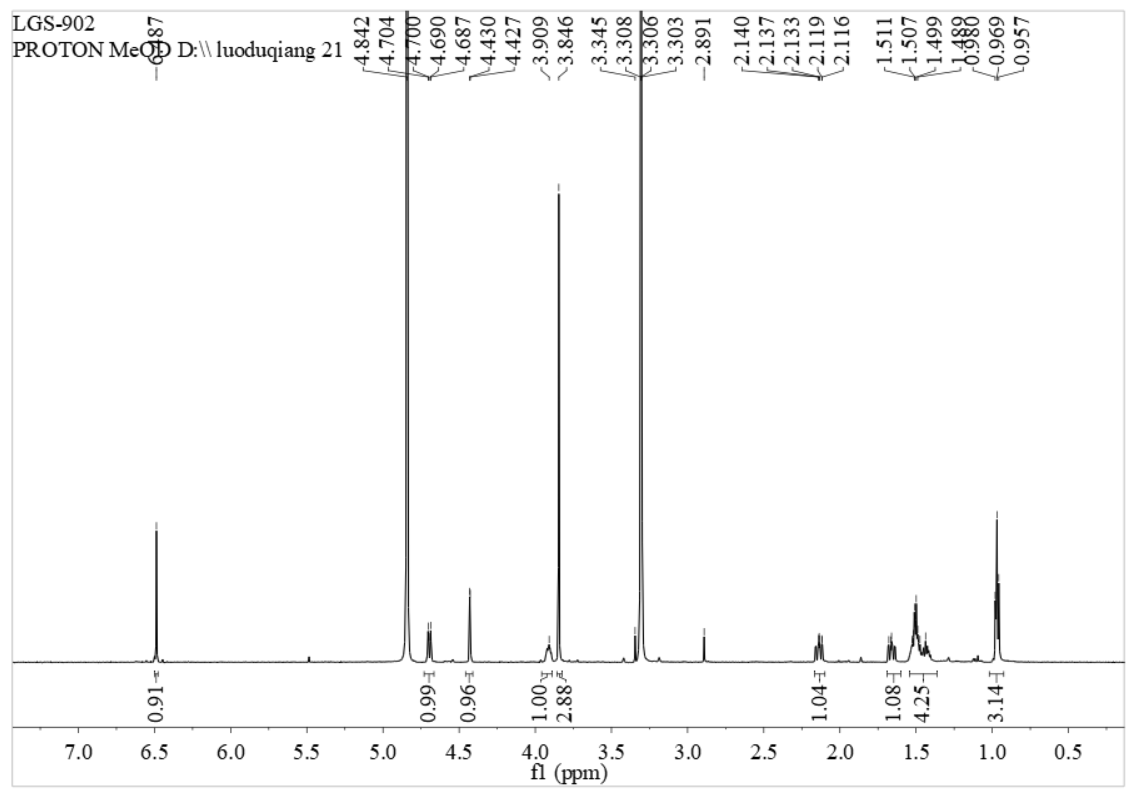


**Figure S1.** ^1^H NMR (600 MHz, CD_3_OD) spectrum of compound **1**
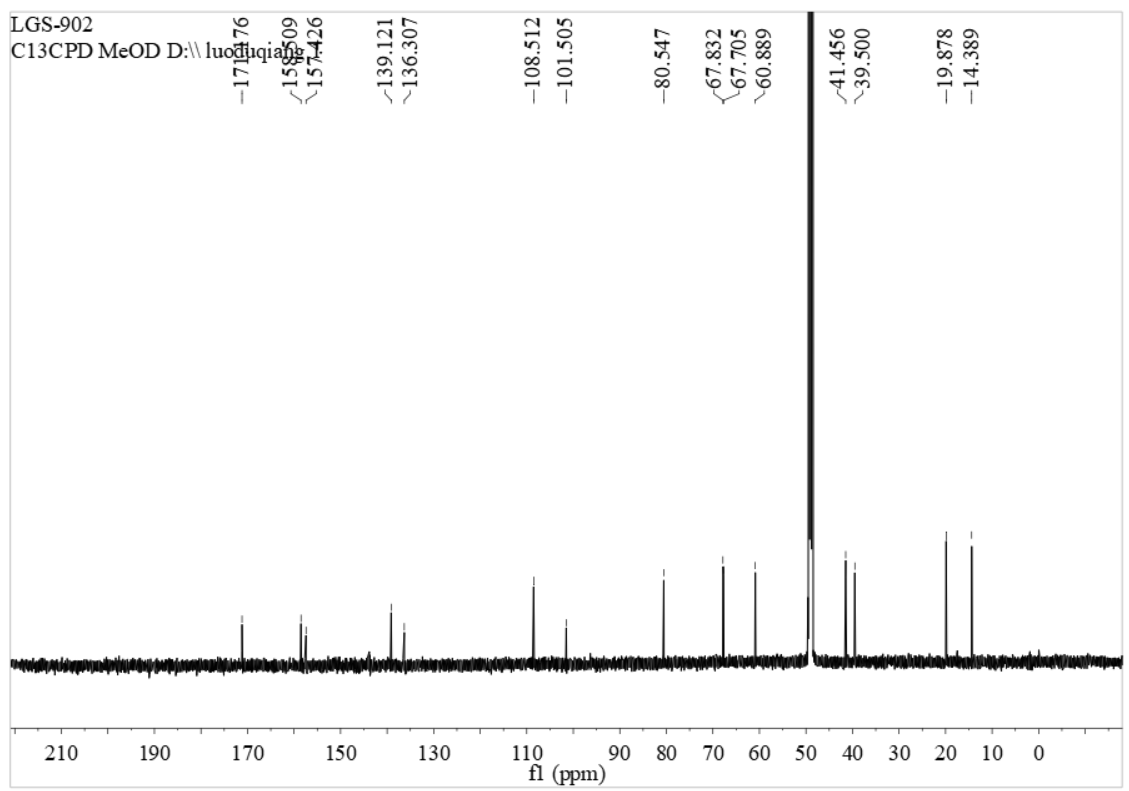


**Figure S2.** ^13^C NMR (150 MHz, CD_3_OD) spectrum of compound **1**


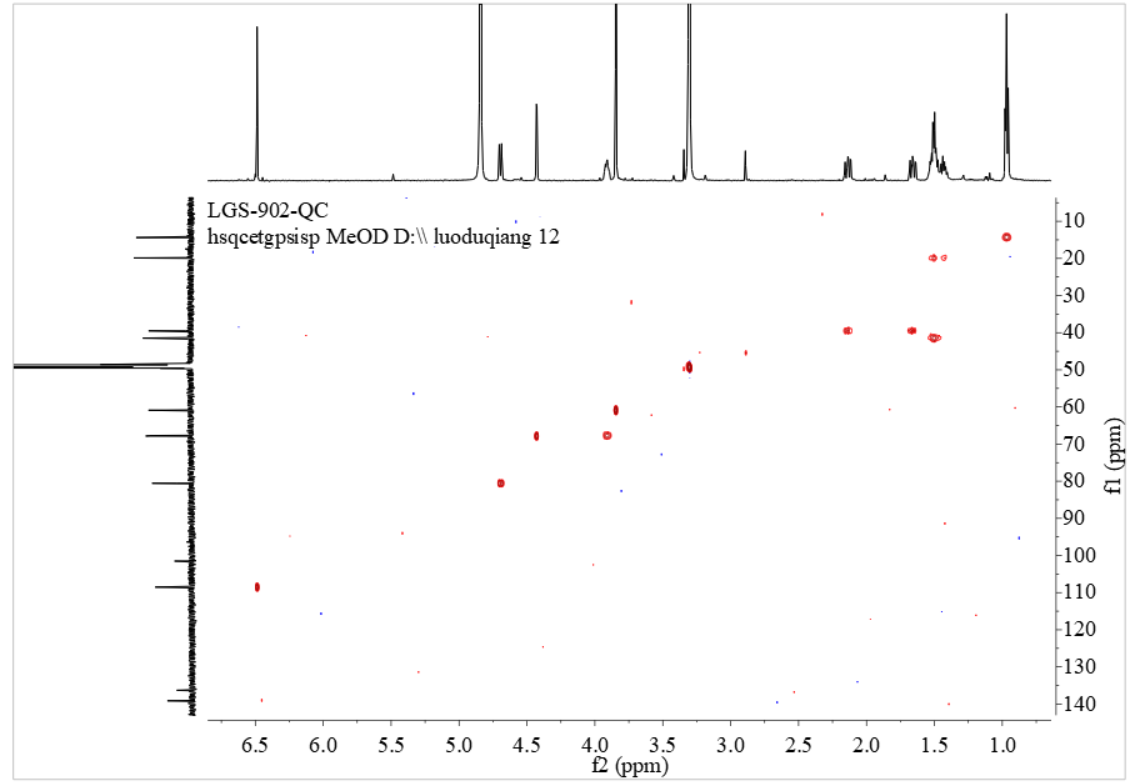


**Figure S3.** HSQC (CD_3_OD) spectrum of compound **1**


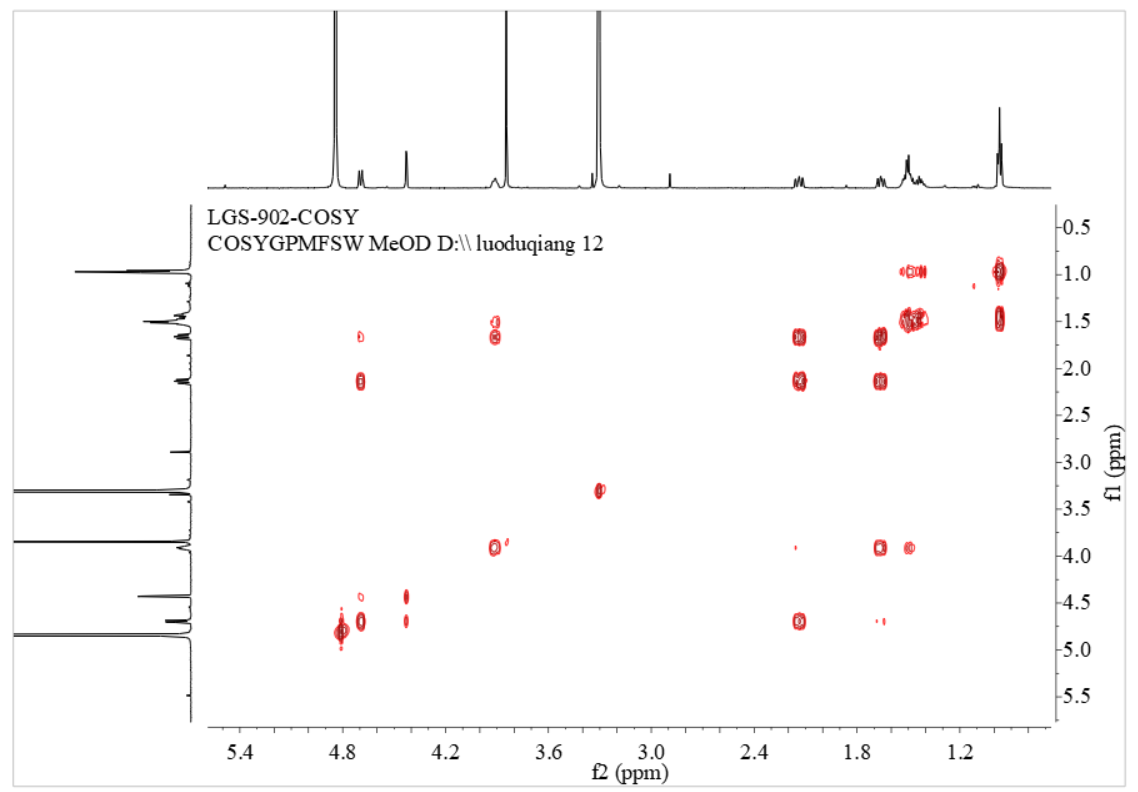


**Figure S4.** COSY (CD_3_OD) spectrum of compound **1**


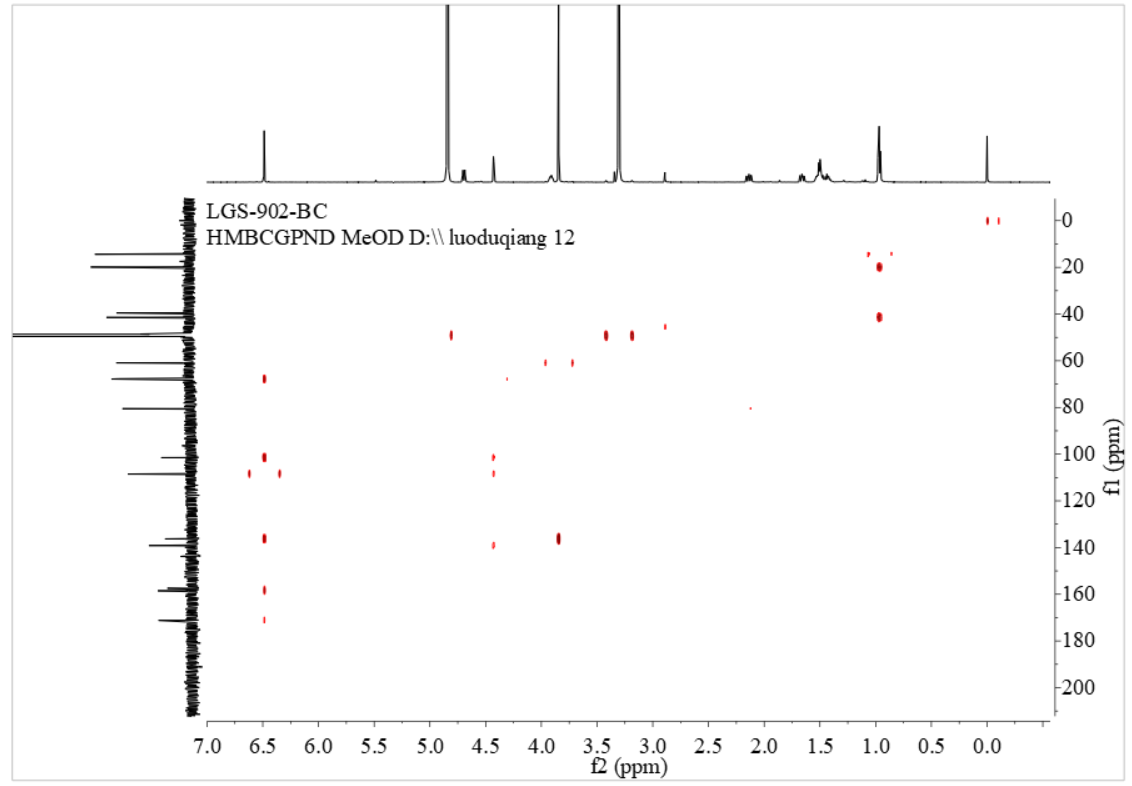


**Figure S5.** HMBC (CD_3_OD) spectrum of compound **1**


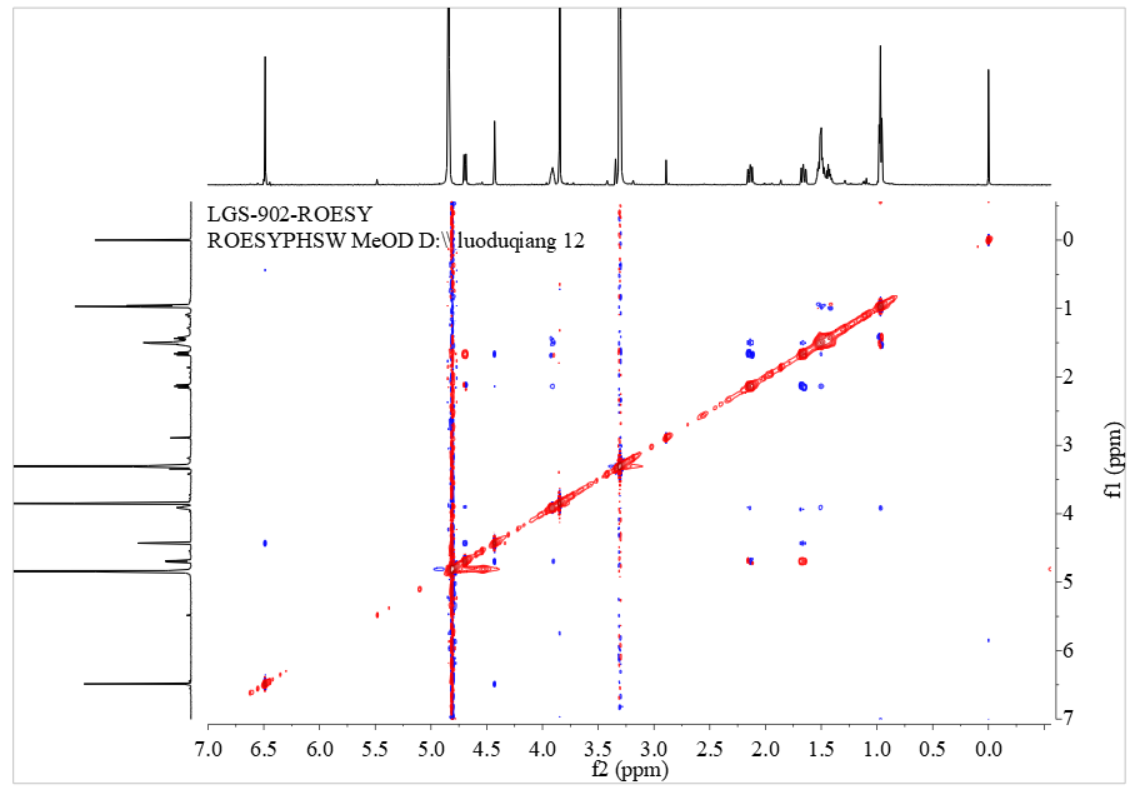


**Figure S6.** NOESY (CD_3_OD) spectrum of compound **1**


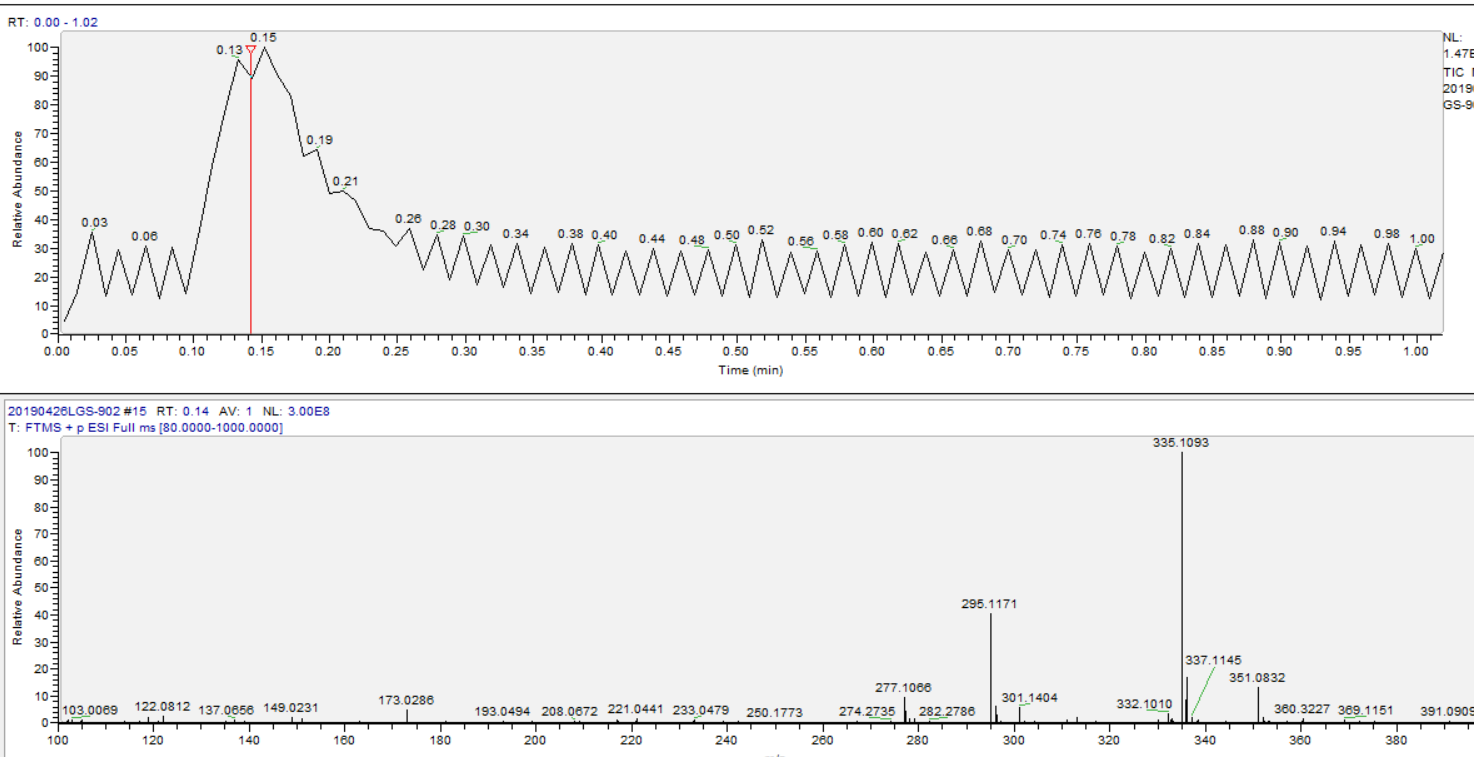


**Figure S7.** HRESIMS spectrum of compound **1**


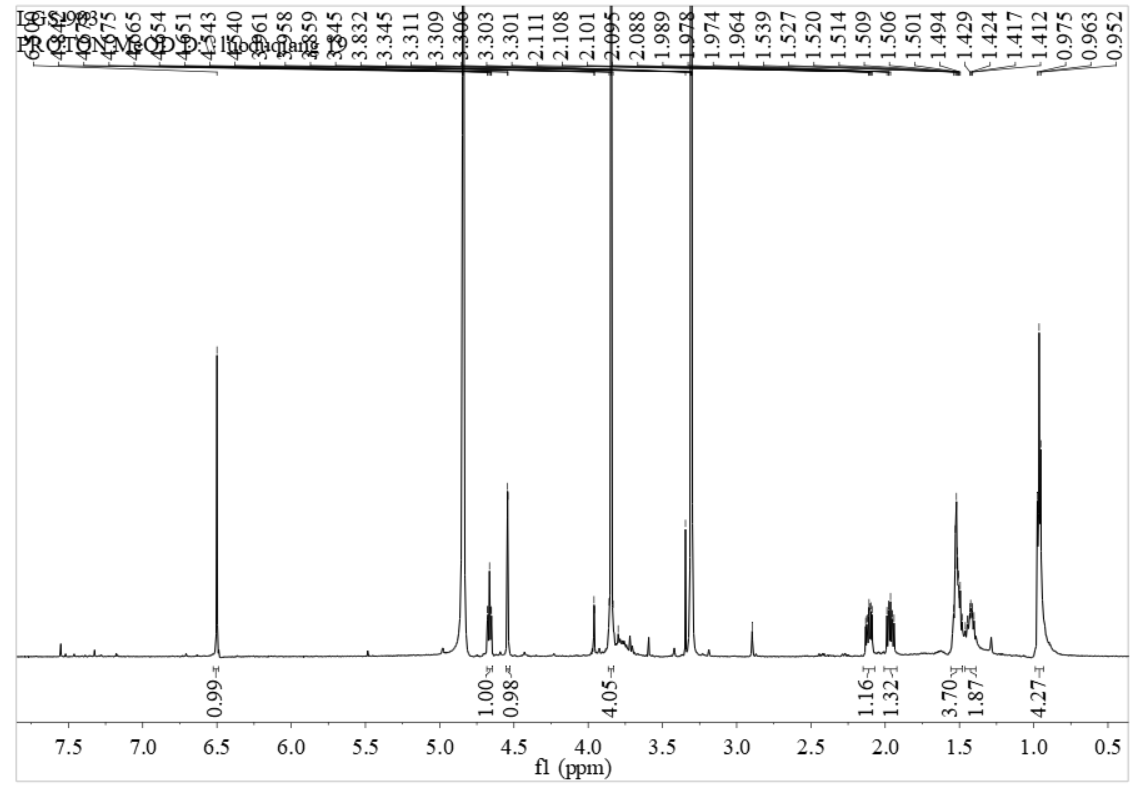


**Figure S8.** ^1^H NMR (600 MHz, CD_3_OD) spectrum of compound **2**


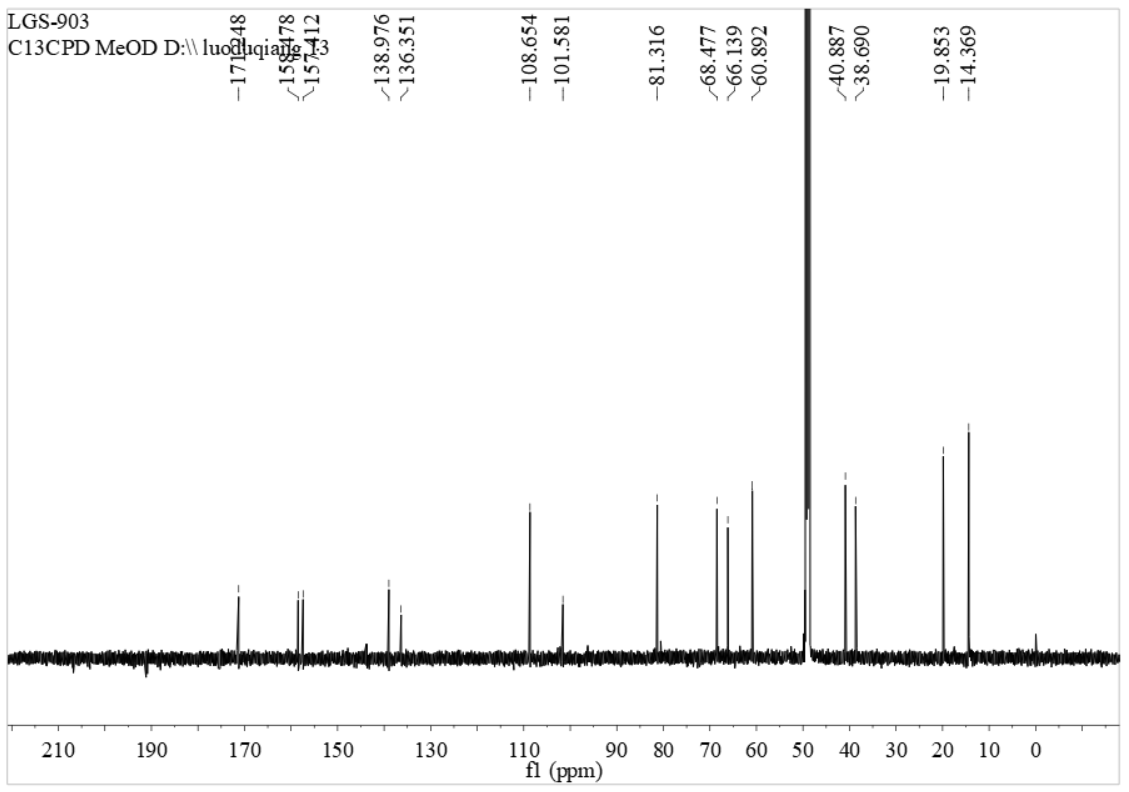


**Figure S9.** ^13^C NMR (150 MHz, CD_3_OD) spectrum of compound **2**


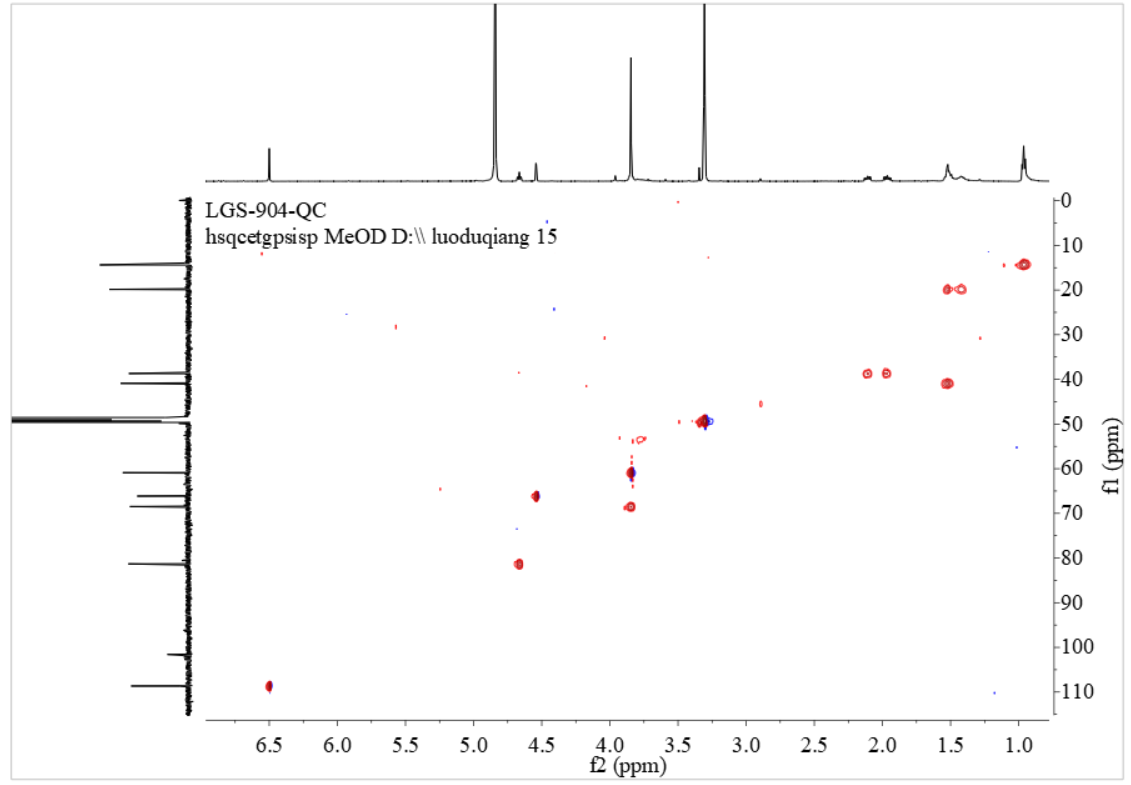


**Figure S10.** HSQC (CD_3_OD) spectrum of compound **2**


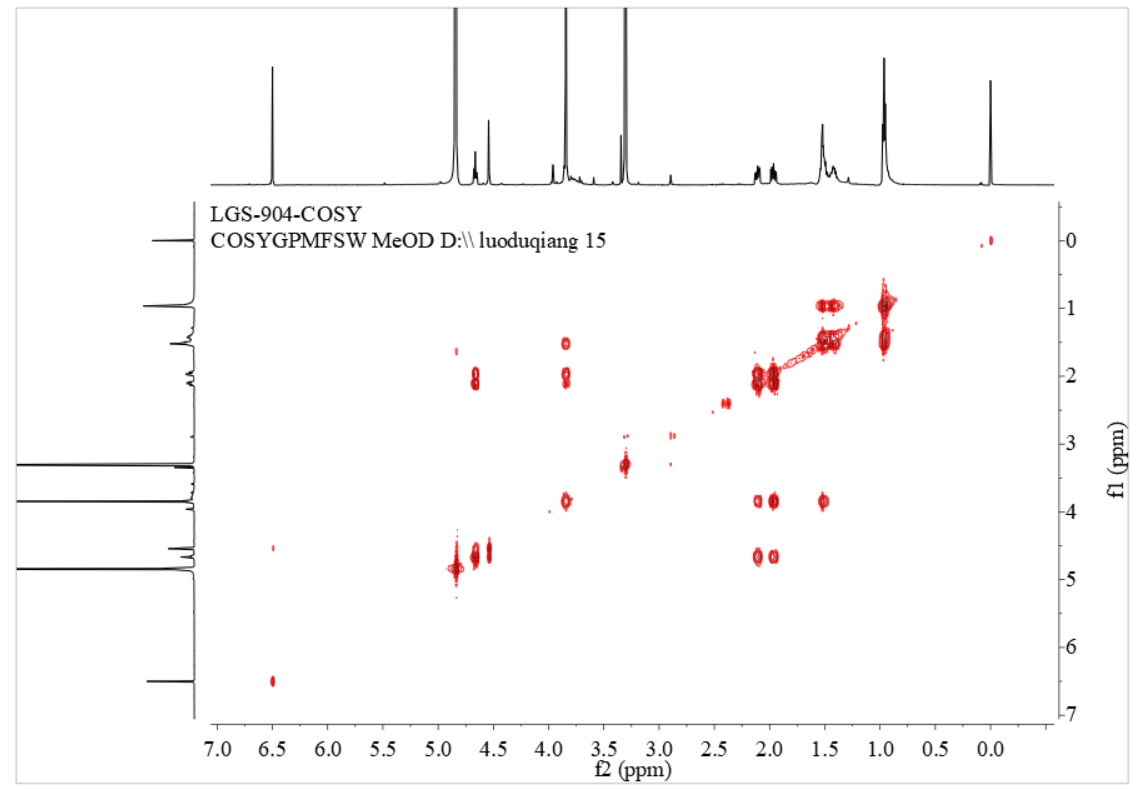


**Figure S11.** COSY (CD_3_OD) spectrum of compound **2**


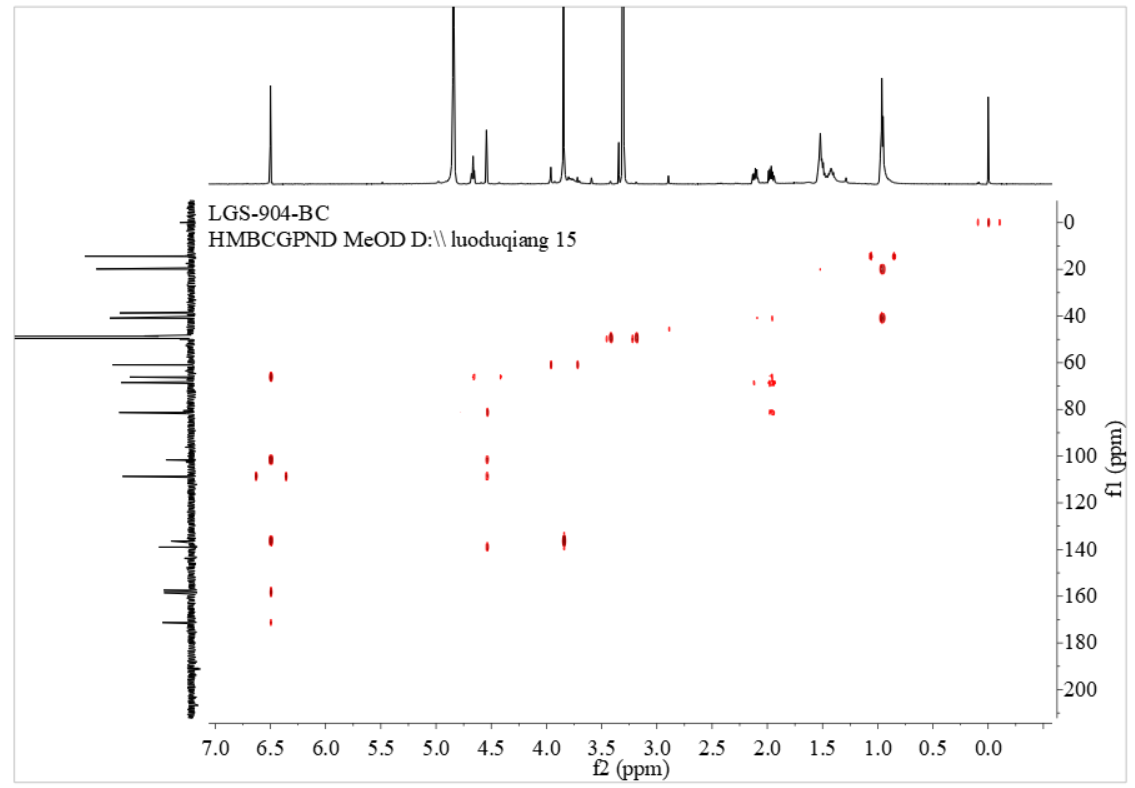


**Figure S12.** HMBC (CD_3_OD) spectrum of compound **2**


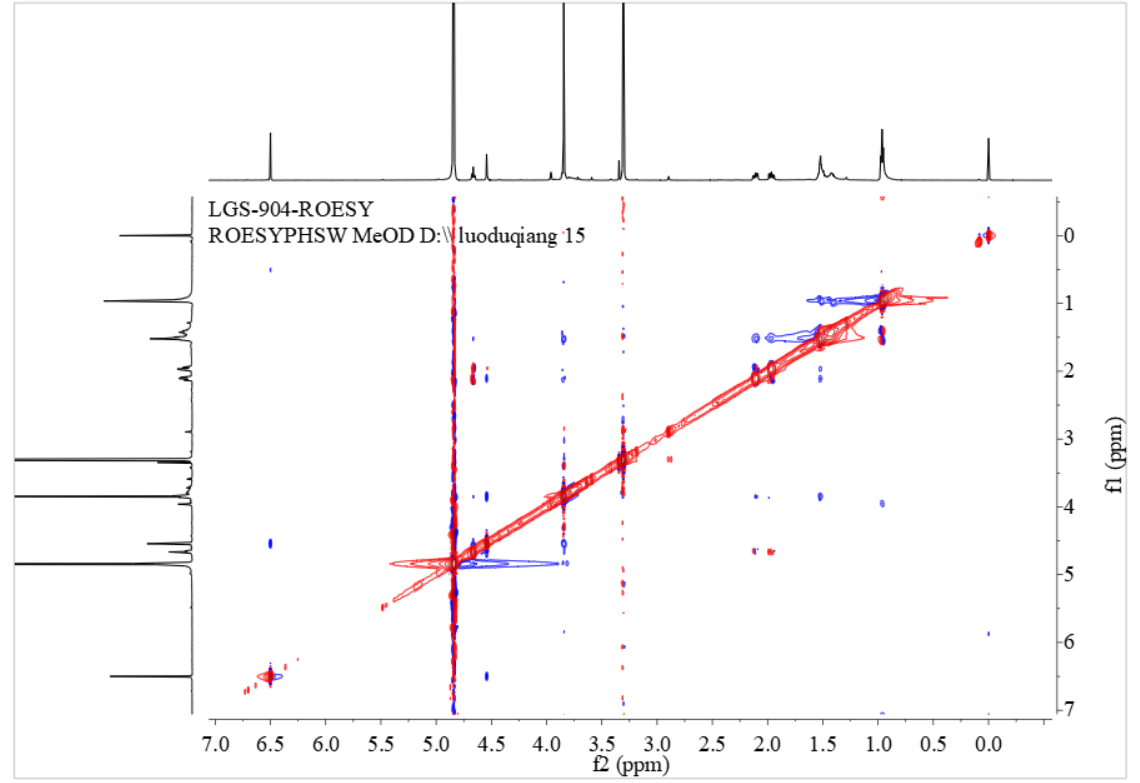


**Figure S13.** NOESY (CD_3_OD) spectrum of compound **2**


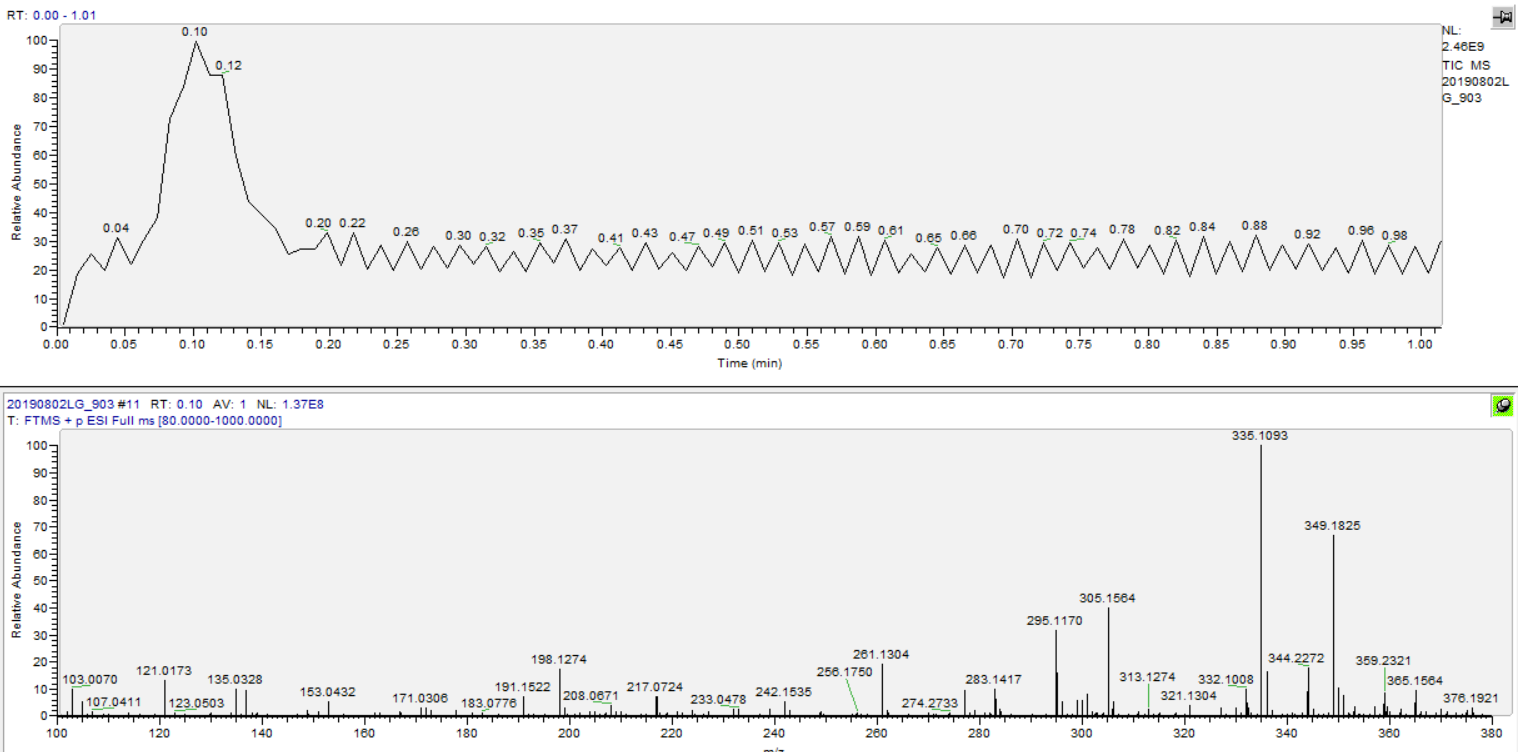


**Figure S14.** HRESIMS spectrum of compound **2**


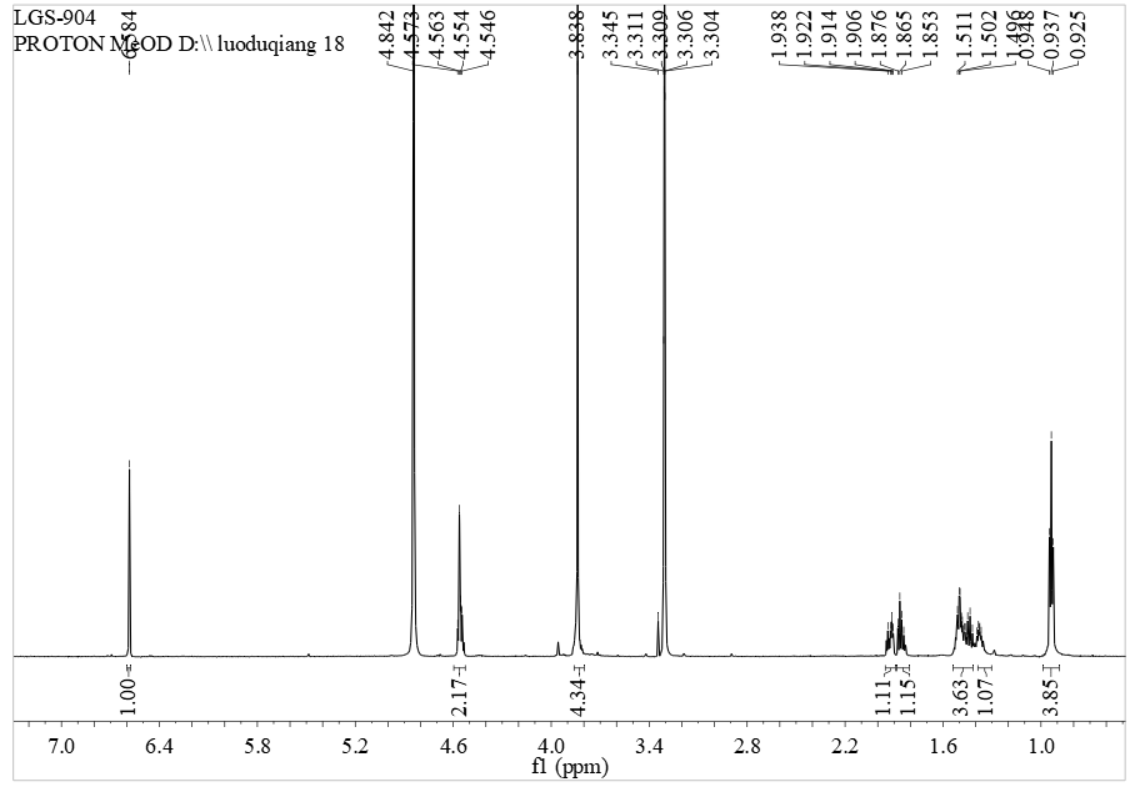


**Figure S15.** ^1^H NMR (600 MHz, CD_3_OD) spectrum of compound **3**


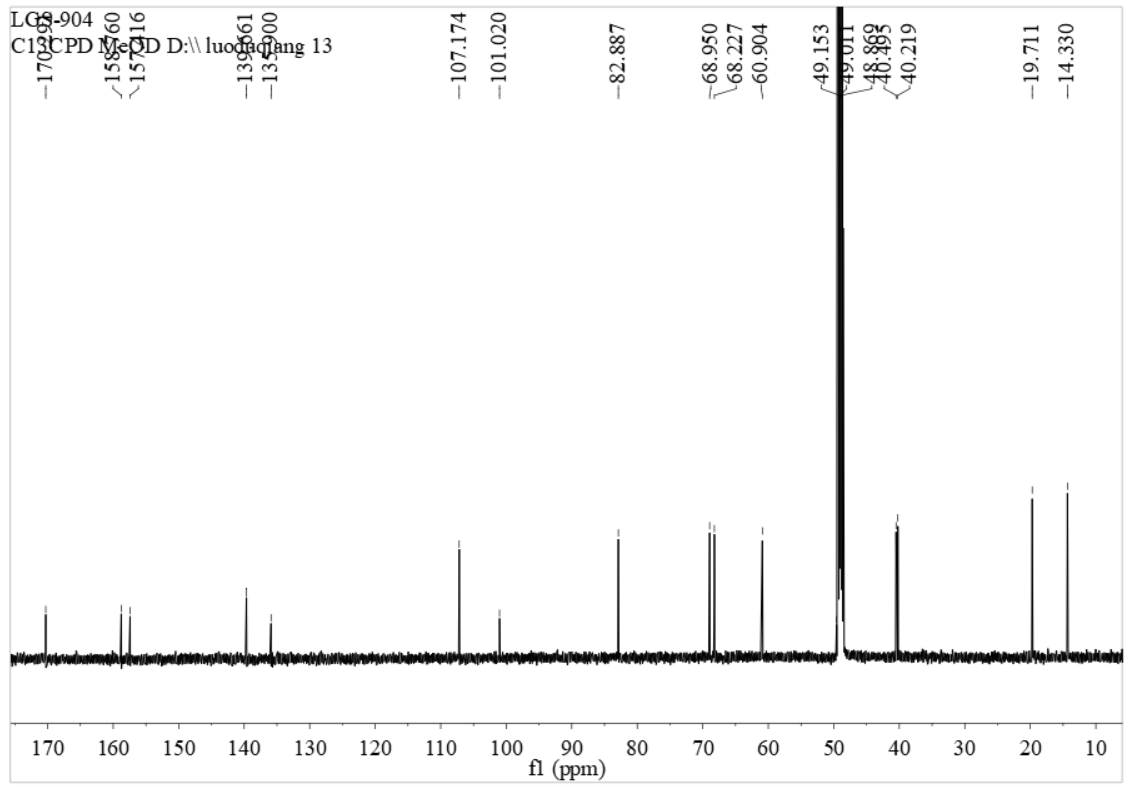


**Figure S16.** ^13^C NMR (150 MHz, CD_3_OD) spectrum of compound **3**


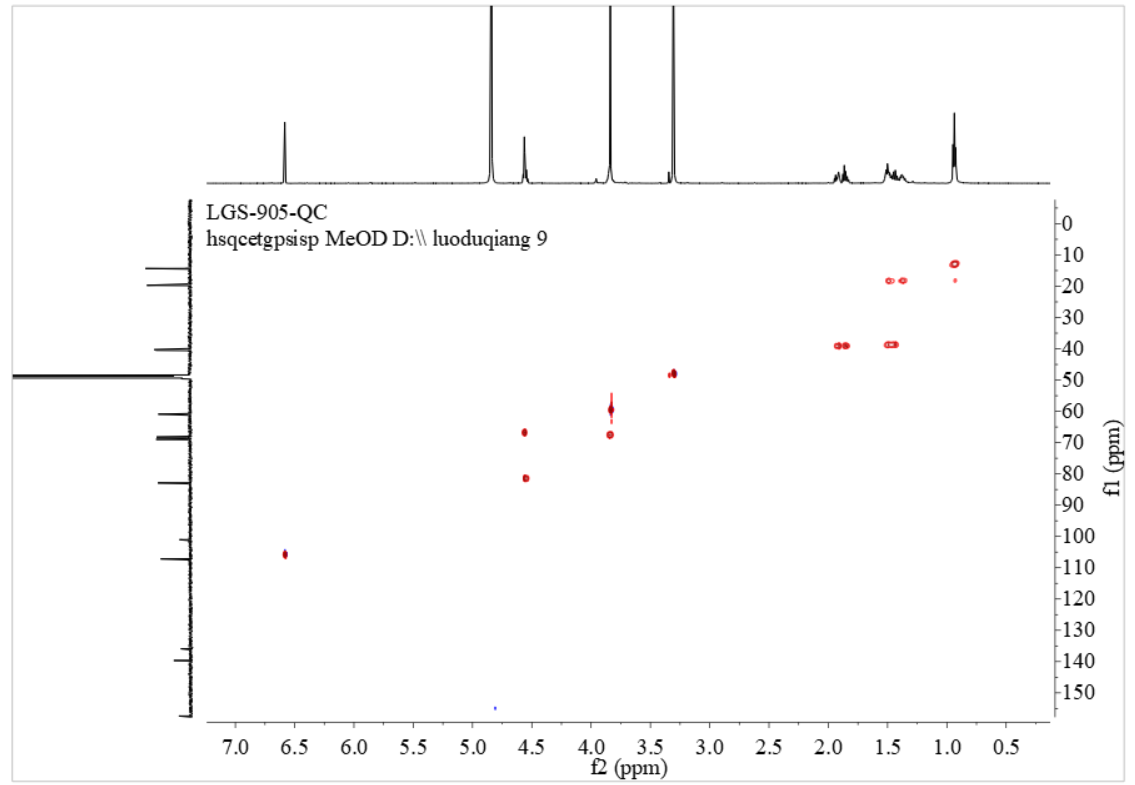


**Figure S17.** HSQC (CD_3_OD) spectrum of compound **3**


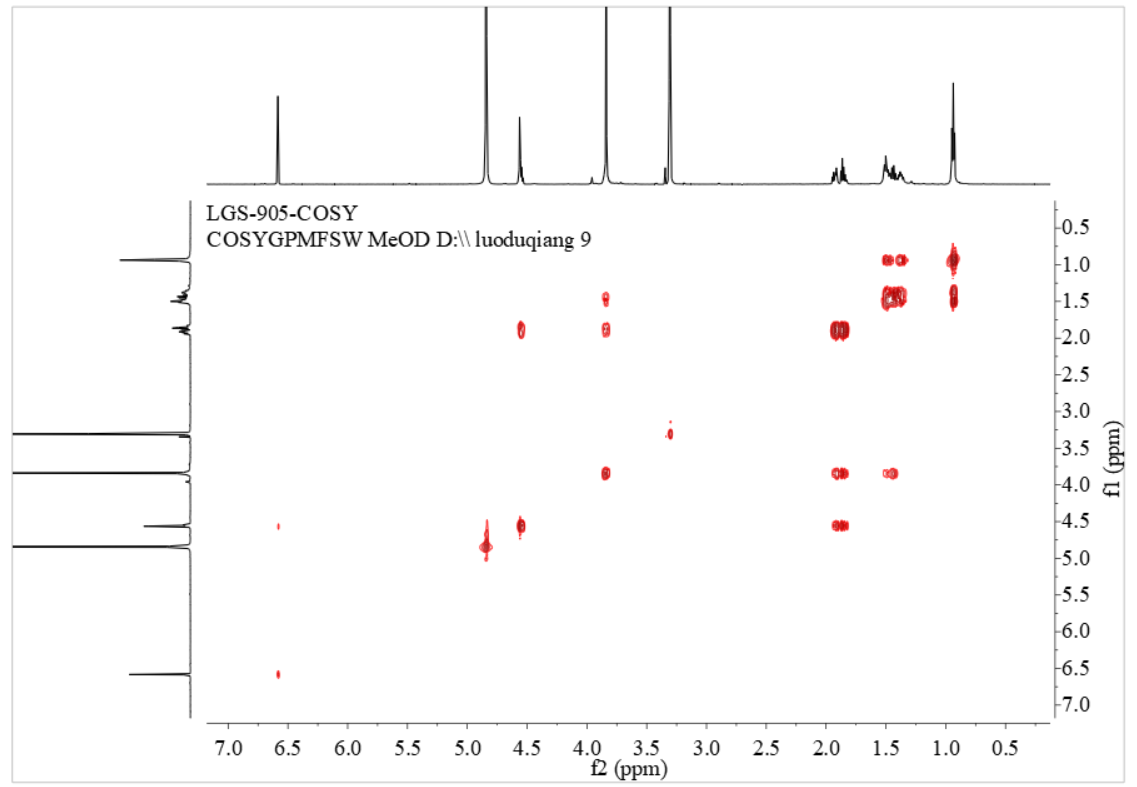


**Figure S18.** COSY (CD_3_OD) spectrum of compound **3**


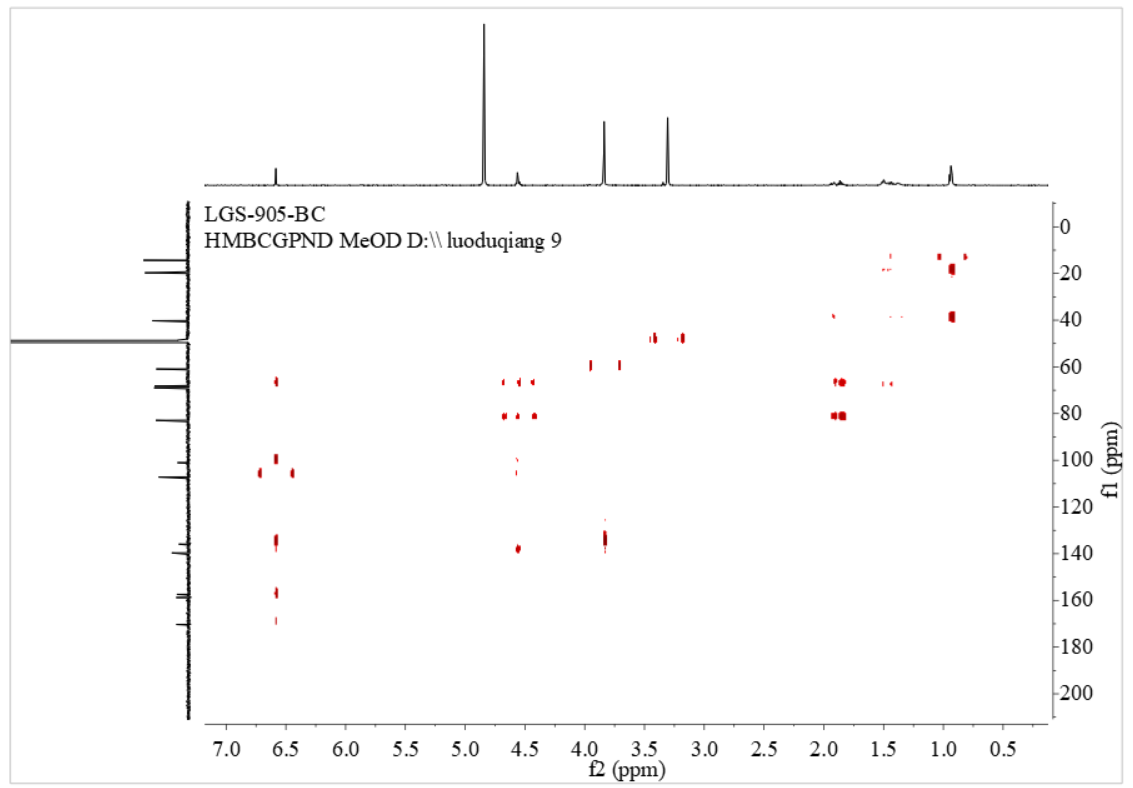


**Figure S19.** HMBC (CD_3_OD) spectrum of compound **3**


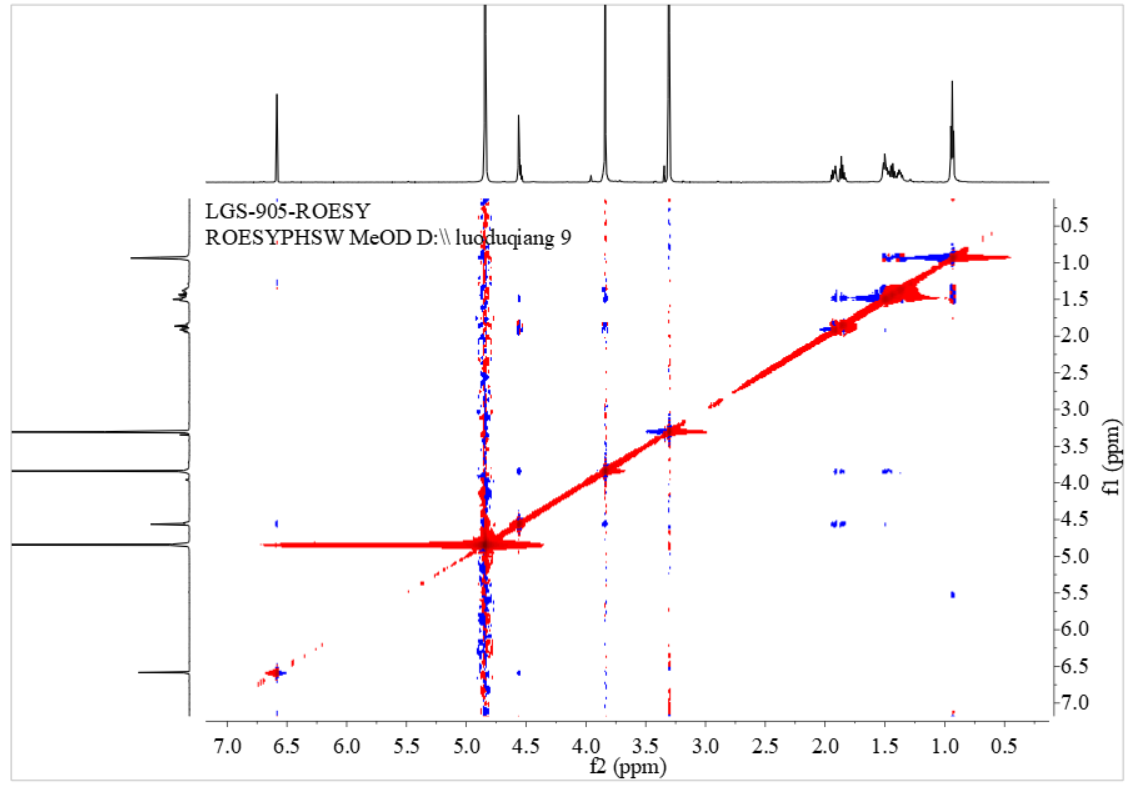


**Figure S20.** NOESY (CD_3_OD) spectrum of compound **3**


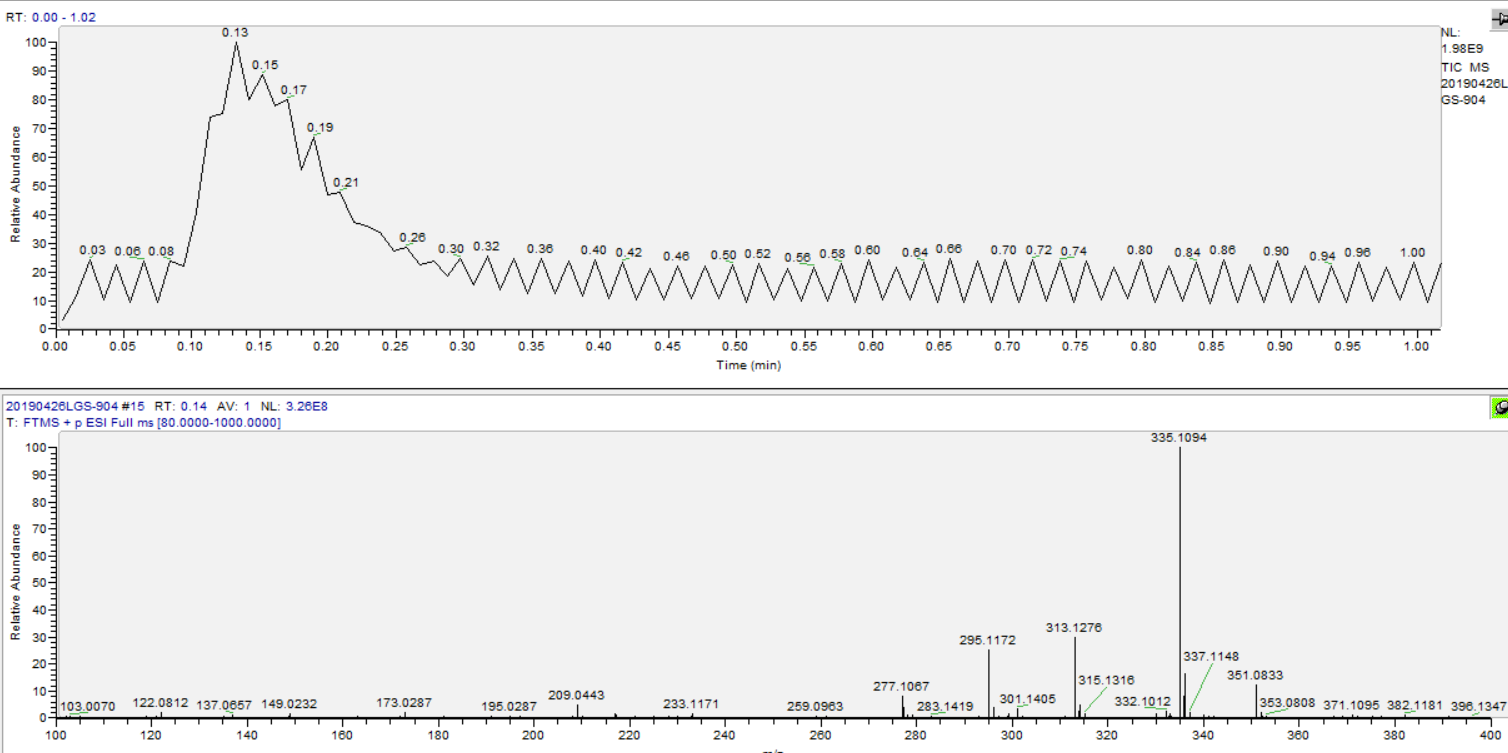


**Figure S21.** HRESIMS spectrum of compound **3**


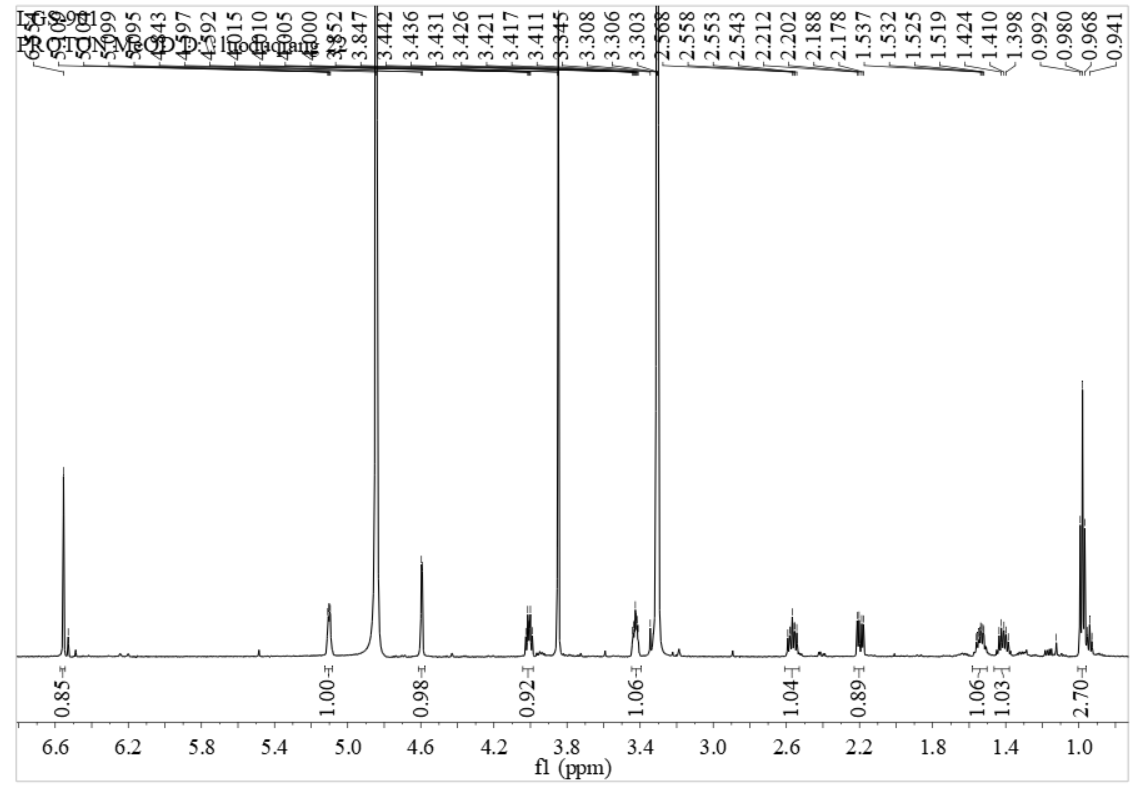


**Figure S22.** ^1^H NMR (600 MHz, CD_3_OD) spectrum of compound **4**


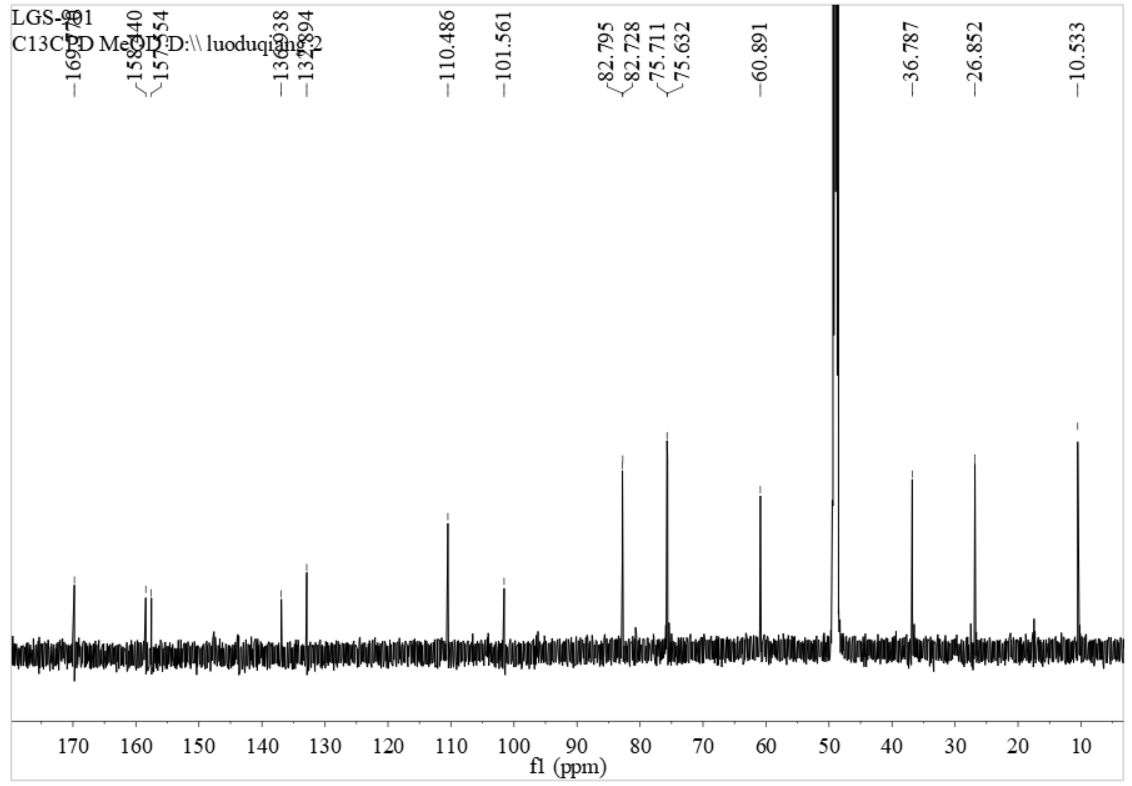


**Figure S23.** ^13^C NMR (150 MHz, CD_3_OD) spectrum of compound **4**


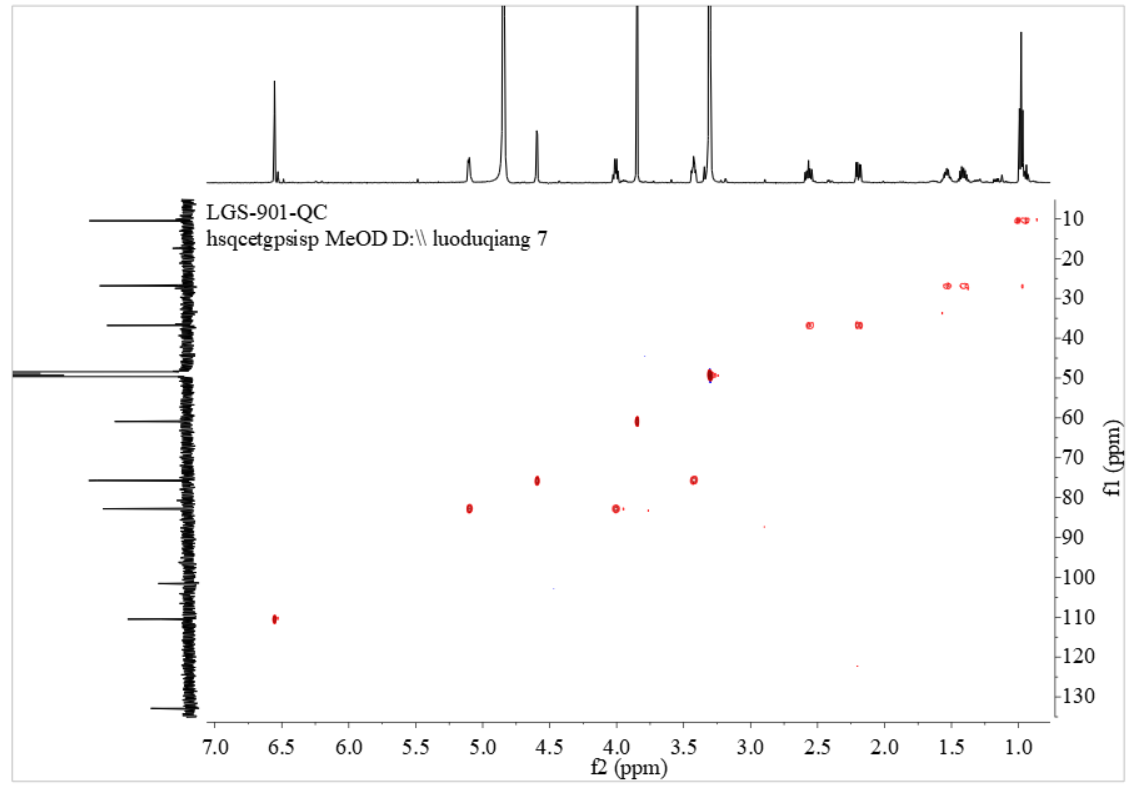


**Figure S24.** HSQC (CD_3_OD) spectrum of compound **4**


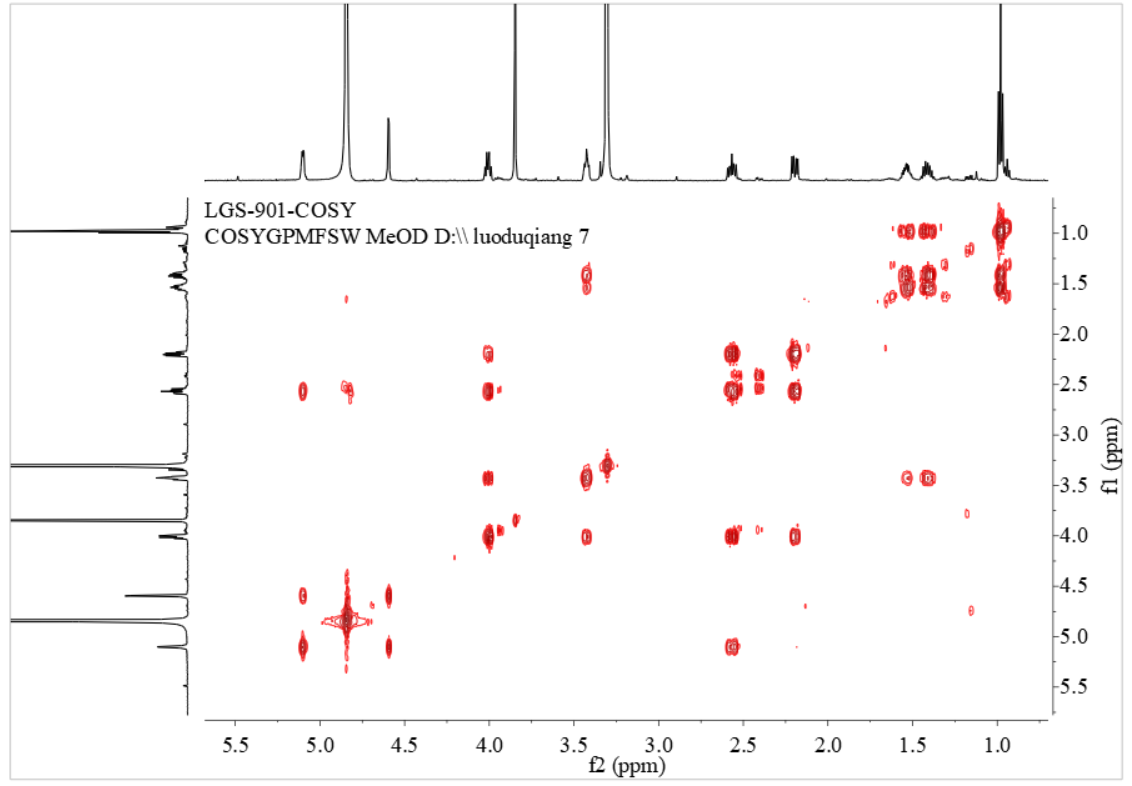


**Figure S25.** COSY (CD_3_OD) spectrum of compound **4**


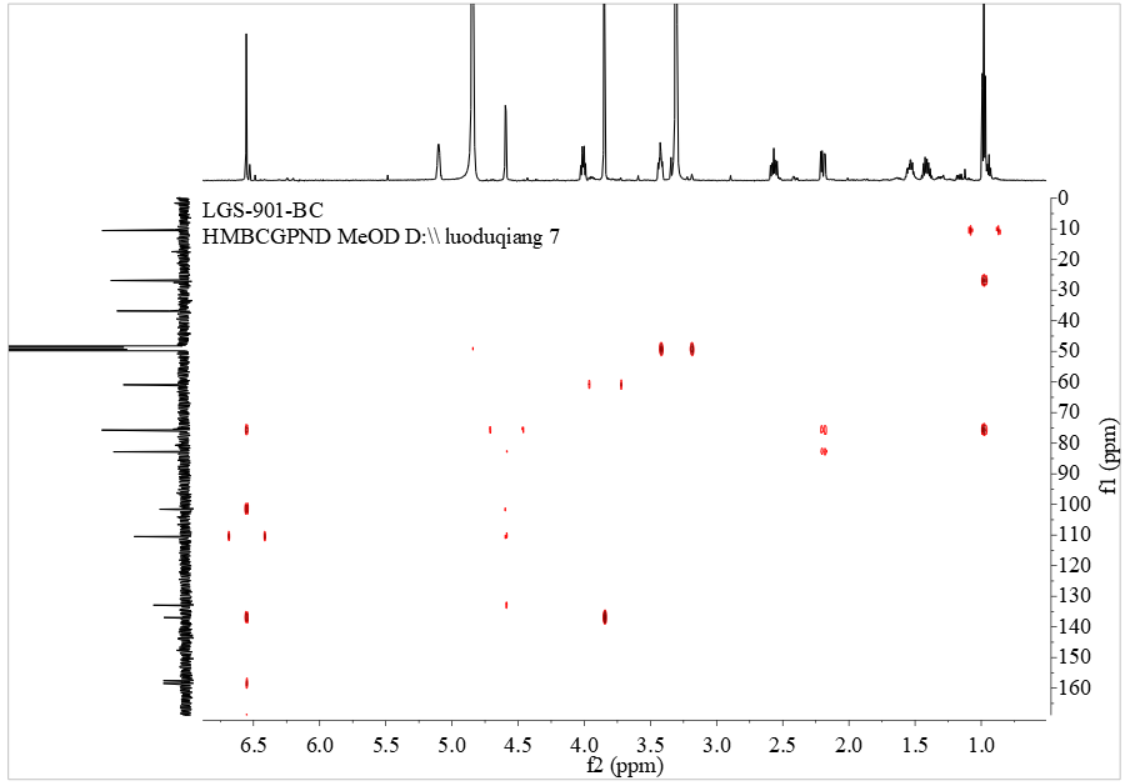


**Figure S26.** HMBC (CD_3_OD) spectrum of compound **4**


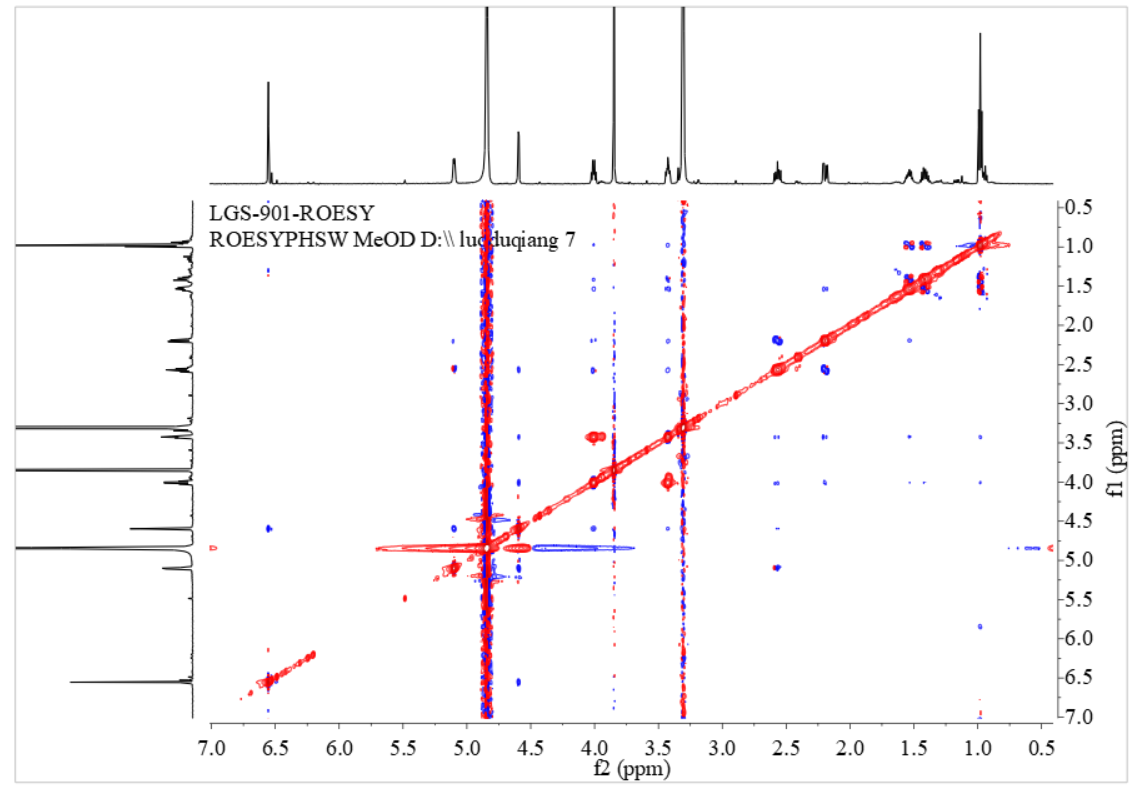


**Figure S27.** NOESY (CD_3_OD) spectrum of compound **4**


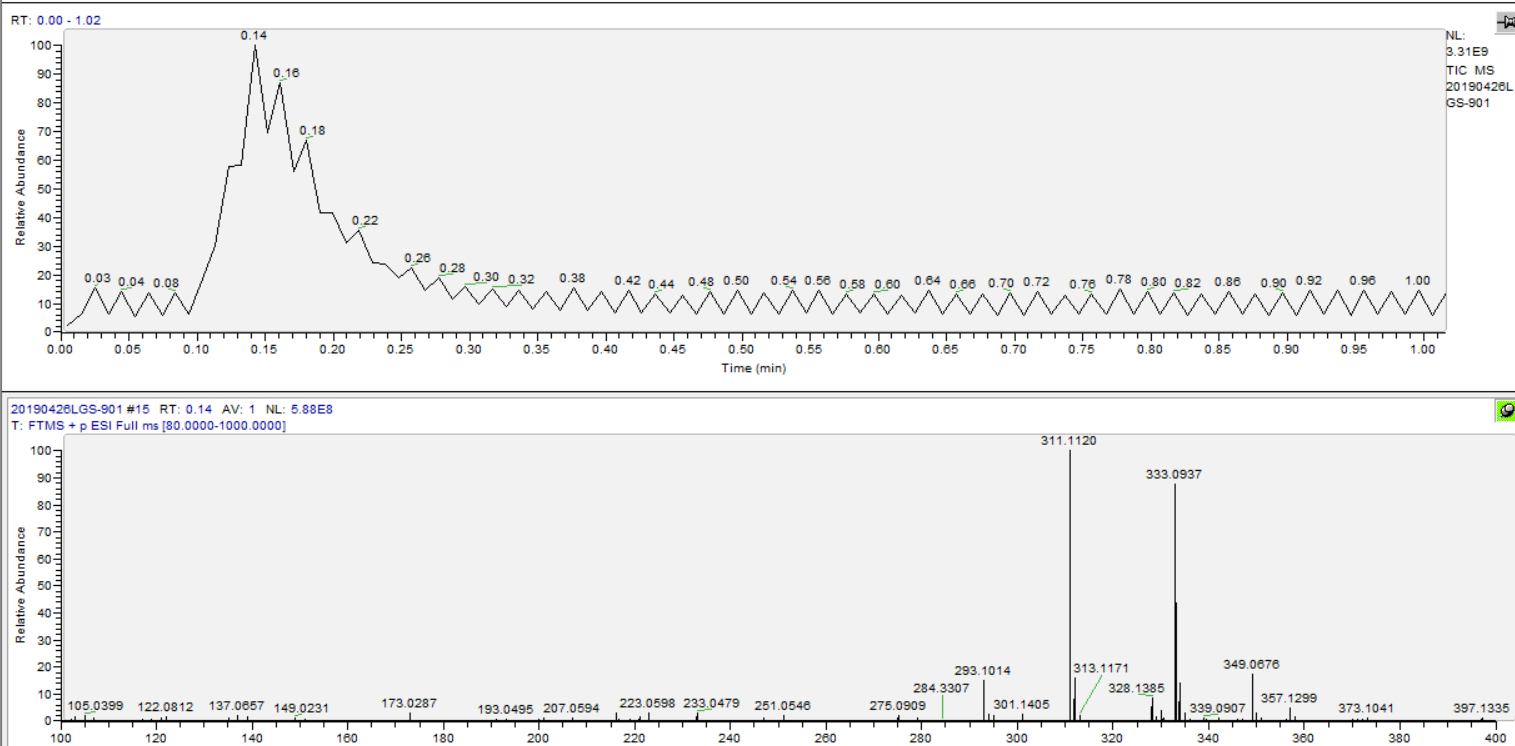


**Figure S28.** HRESIMS spectrum of compound **4**

**Table S1.** The coordinate for the lowest-energy conformer (**1**) in ^13^C NMR calculations

----------------------------------------------------------------------------------

Coordinates (Angstroms)

X Y Z

-----------------------------------------------------------------------------------

C 3.38203000 -0.73544500 -0.32285000

C 2.60441600 -1.82201100 0.09500600

C 1.25624700 -1.66180500 0.42380300

C 0.67735000 -0.41001400 0.31927700

C 1.42838400 0.69331500 -0.12677200

C 2.80174000 0.53060800 -0.44793000

C -0.76034000 -0.19284900 0.72682700

C -1.36505800 0.97555300 -0.06339900

O -0.50349700 2.14553900 -0.07353200

C 0.82310300 2.01982100 -0.25124600

C -2.69595700 1.51815100 0.46304900

O 1.46550100 3.03310200 -0.50413200

O 3.57325100 1.54487400 -0.87456300

O 4.68566800 -1.02409700 -0.65780600

O 3.17119800 -3.04690000 0.18203900

C 5.71525100 -0.30710200 0.04835300

C -3.86927900 0.54423300 0.57587300

O -3.55974100 -0.36931000 1.65279600

C -4.21808100 -0.19472100 -0.72068500

C -5.50060500 -1.03158000 -0.64124200

C -5.86961300 -1.68493400 -1.97530000

O -0.80558300 -0.00210200 2.13451600

H 0.69248300 -2.51908500 0.77289400

H -1.33895600 -1.08551500 0.46218800

H -1.46416000 0.65365900 -1.10713700

H -2.99412000 2.34040400 -0.19370100

H -2.51433900 1.96051800 1.44572500

H 3.02858100 2.36625700 -0.84356200

H 4.08818500 -2.96639600 -0.12727500

H 6.65608600 -0.75886900 -0.26255800

H 5.69842300 0.74835200 -0.21702400

H 5.58738700 -0.42435300 1.12870800

H -4.74163100 1.13916300 0.88039400

H -4.32218500 -0.92392200 1.85155800

H -4.32909200 0.55369200 -1.51546100

H -3.38139700 -0.83847100 -1.01436100

H -5.38911900 -1.82020500 0.11434500

H -6.32960400 -0.39645600 -0.30385000

H -6.78245500 -2.28004300 -1.88861900

H -6.03804400 -0.93344000 -2.75258900

H -5.07452400 -2.34943600 -2.32682800

H -1.72342400 -0.16203600 2.40602400

-----------------------------------------------------------------------------------

**Table S2.** The coordinate for the lowest-energy conformer (**2**) in ^13^C NMR calculations

----------------------------------------------------------------------------------

Coordinates (Angstroms)

X Y Z

-----------------------------------------------------------------------------------

C 3.72392000 -0.31412300 -0.18560900

C 3.15152700 -1.59148000 -0.20769700

C 1.76651900 -1.76409100 -0.14707800

C 0.94784500 -0.65168000 -0.08204700

C 1.49522500 0.64494100 -0.09144000

C 2.90353600 0.81747600 -0.13792200

C -0.54826300 -0.80221500 0.05273900

C -1.25666200 0.40375900 -0.57605300

O -0.69532300 1.66793300 -0.13656100

C 0.63634700 1.82894600 -0.03908800

C -2.74966800 0.53515100 -0.27247100

O 1.06705600 2.96897700 0.09380900

O 3.48763900 2.02766100 -0.14294100

O 5.09571800 -0.26892000 -0.28981600

O 3.95543600 -2.67604500 -0.28780100

C -3.60867700 -0.67020500 -0.65118000

O -3.36172600 -1.68690900 0.34841500

C -5.10522600 -0.34788000 -0.75366600

C -5.74463200 0.26094200 0.49955400

C -7.25801100 0.43877500 0.35479900

O -0.86006900 -0.98229300 1.42710800

C 5.80874100 0.39452400 0.77056800

H 1.36000500 -2.76866400 -0.13402800

H -0.86624500 -1.68560900 -0.51473900

H -1.09938500 0.34845900 -1.66160500

H -3.11214000 1.41586300 -0.80948500

H -2.86928900 0.74708400 0.79241900

H 2.77532600 2.70195000 -0.04222100

H 4.86896100 -2.35681400 -0.36885800

H -3.28004900 -1.06090100 -1.62550600

H -3.84852000 -2.49020800 0.12992400

H -5.24856900 0.32307900 -1.61018500

H -5.63498200 -1.27426500 -1.01826200

H -5.52840200 -0.37497400 1.36394200

H -5.28634800 1.23211300 0.71393700

H -7.69382800 0.87889100 1.25559800

H -7.50697100 1.09526300 -0.48511800

H -7.75918700 -0.51916100 0.18166400

H -1.75510900 -1.35654900 1.45906600

H 6.86500100 0.22391300 0.56796500

H 5.59237400 1.46124300 0.76896400

H 5.54237700 -0.04098400 1.73836200

-----------------------------------------------------------------------------------
